# Genotypic characterisation of monepantel resistance in historical and newly derived field strains of *Teladorsagia circumcincta*

**DOI:** 10.1016/j.ijpddr.2019.10.002

**Published:** 2019-10-06

**Authors:** Frank Turnbull, Eileen Devaney, Alison A. Morrison, Roz Laing, Dave J. Bartley

**Affiliations:** a Institute of Biodiversity Animal Health and Comparative Medicine, University of Glasgow, Bearsden Road, Glasgow, G61 1QH, United Kingdom; b Moredun Research Institute, Pentlands Science Park, Bush Loan, Edinburgh, EH26 0PZ, United Kingdom

**Keywords:** Anthelmintic resistance, Monepantel, *Teladorsagia circumcincta*, MPTL-1 gene, Microsatellite

## Abstract

Recent reports of monepantel (MPTL) resistance in UK field isolates of *Teladorsagia circumcincta* has highlighted the need for a better understanding of the mechanism of MPTL-resistance in order to preserve its anthelmintic efficacy in this economically important species.

Nine discrete populations of *T. circumcincta* were genotypically characterised; three MPTL-susceptible isolates, three experimentally selected MPTL-resistant strains and three field derived populations. Full-length *Tci-mptl-1* gene sequences were generated and comparisons between the MPTL-susceptible isolates, MPTL-resistant strains and one field isolate, showed that different putative MPTL-resistance conferring mutations were present in different resistant isolates. Truncated forms of the *Tci-mptl-1* gene were also observed. The genetic variability of individual larvae, within and between populations, was examined using microsatellite analyses at 10 ‘neutral’ loci (presumed to be unaffected by MPTL). Results confirmed that there was little background genetic variation between the populations, global *F*_ST_ <0.038. Polymorphisms present in exons 7 and 8 of *Tci-mptl-1* enabled genotyping of individual larvae. A reduction in the number of genotypes was observed in all MPTL-resistant strains compared to the MPTL-susceptible strains that they were derived from, suggesting there was purifying selection at *Tci-mptl-1* as a result of MPTL-treatment. The potential link between benzimidazole (BZ)-resistance and MPTL-resistance was examined by screening individual larvae for the presence of three SNPs associated with BZ-resistance in the β-tubulin isotype-1 gene. The majority of larvae were BZ-susceptible homozygotes at positions 167 and 198. Increased heterozygosity at position 200 was observed in the MPTL-resistant strains compared to their respective MPTL-susceptible population. There was no decrease in the occurrence of BZ-resistant genotypes in larvae from each population.

These differences, in light of the purifying selection at this locus in all MPTL-resistant isolates, suggests that *Tci-mptl-1* confers MPTL-resistance in *T. circumcincta*, as in *Haemonchus contortus*, but that different mutations in *Tci-mptl-1* can confer resistance in different populations.

## Highlights

• Different null mutations of *Tci-mptl-1* are present in monepantel resistant strains.
• Microsatellite analysis showed little divergence between UK Teladorsagia isolates.
• *Tci-mptl-1* appears to be under purifying selection after monepantel administration.
• These findings are consistent with monepantel-resistance in Haemonchus.
• Monepantel treatment may select against benzimidazole-resistance.

## Graphical abstract

**Figure:**
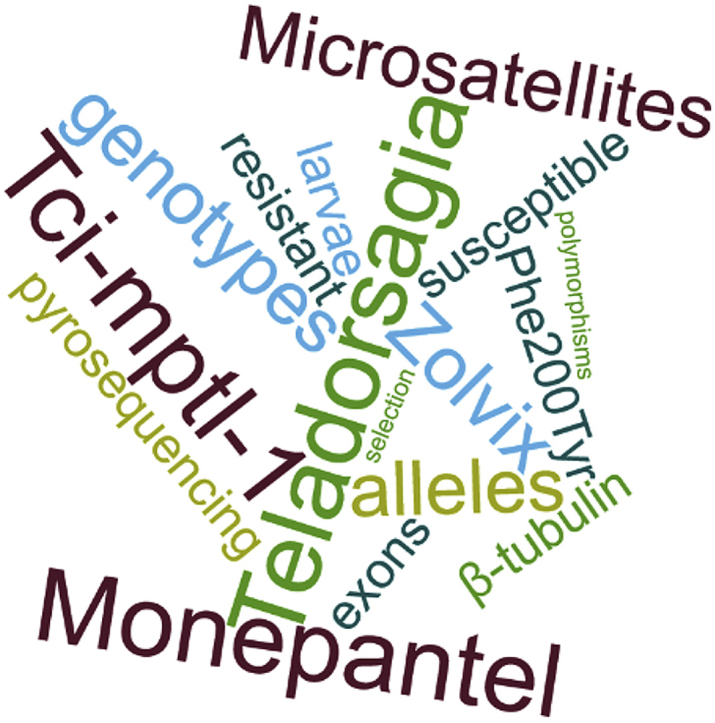

## 1 Introduction

*Teladorsagia circumcincta* is the most prevalent parasitic nematode of sheep in temperate regions (Stear et al., 2009), and is a common cause of parasitic gastroenteritis. Effective control of *T. circumcincta* has relied heavily on the use of broad spectrum anthelmintic drugs, but this has led to the emergence of drug resistance in this species which is now a major worldwide issue (Kaplan, 2004; Jabbar et al., 2006). Currently there are five broad-spectrum classes of anthelmintics available on the UK market, these being the benzimidazoles (BZ), an imidazothiazole i.e. levamisole (LEV), the macrocyclic lactones, such as ivermectin (IVM) and moxidectin (MOX), an amino-acetonitrile derivative, i.e. monepantel (MPTL) and a spiroindole, i.e. derquantel.

Monepantel was released under the trade name Zolvix® (Novartis Animal Health) initially in New Zealand in 2009, followed by release in 2010 to the Australian and UK markets (Hosking et al., 2010). Unfortunately, MPTL resistance has developed quickly, often within 2–3 years of first use. The first cases of resistance were highlighted in field isolates of *T. circumcincta* and *Trichostrongylus colubriformis* in New Zealand flocks of sheep and goats (Leathwick et al., 2013; Scott et al., 2013). Resistance to MPTL has since also been reported in *Haemonchus contortus* from sheep in Uruguay (Mederos et al., 2014), the Netherlands (Van den Brom et al., 2015), Australia (Sales and Love, 2016) and Brazil (Martins et al., 2017; Ramos et al., 2018), in *T. colubriformis* in Brazilian sheep and goat flocks (Cintra et al., 2016, 2018) and in *Trichostrongylus vitrinus*, *T. circumcincta* and *Oesophagostomum venulosum* from a UK flock (Hamer et al., 2018; Bartley et al., 2019).

Prior to the release of MPTL to market, a number of studies were carried out using *H. contortus* and the free-living nematode *Caenorhabditis elegans* (Kaminsky et al., 2008; Rufener et al., 2009) to determine its likely mode of action and to identify mutations that may correlate with resistance to it. These studies identified a nematode-specific nicotine acetylcholine receptor (nAChR) subunit (originally known as *acr-23*, now called *mptl-1*), belonging to the DEG-3 family, as the target of MPTL. MPTL was shown to bind to its receptor resulting in the constant, uncontrolled flux of Na^+^ and K^+^ cations which leads to the depolarisation of muscle cells and causes irreversible paralysis in the nematode (Baur et al., 2015). Two functional copies of the *C. elegans acr-23* gene, the gene with the highest similarity to *mptl-1* in *H. contortus*, appear to be required for full sensitivity to MPTL and complementation studies, in which the addition of extra copies of the wild-type *acr-23* gene were shown to restore sensitivity to the drug (Rufener et al., 2013). These studies have provided supporting evidence that *mptl-1* is the major target of MPTL in the parasitic nematode *H. contortus*. Sequencing of *mptl-1* from drug sensitive and resistant strains of *H. contortus* has identified a large number of mutations that result in defects in splicing and the introduction of premature stop codons in the resistant strains (Rufener et al., 2009). In addition, levels of expression of *mptl-1*, *des-2* and *deg-3* (all members of the DEG-3 family) were found to be significantly altered in resistant versus susceptible isolates. Since these initial studies, work to identify mechanisms of MPTL-resistance in field isolates of *H. contortus* has continued. For example, differences in expression of drug transporters between resistant and susceptible worms have been identified (Raza et al., 2016a), while additional analysis using a larval development test have suggested that at least two separate mechanisms of MPTL resistance are at play in *H. contortus* (Raza et al., 2016b). These studies imply that resistance to MPTL is a complex trait. More recent work on field-derived isolates of *H. contortus* has confirmed the importance of MPTL-1 and again demonstrated the presence of multiple mutations in the *mptl-1* gene from resistant parasites (Bagnall et al., 2017).

In most cases the parasite populations reported to have developed resistance to MPTL were already resistant to one or more of the three older broad-spectrum anthelmintic classes (BZs, macrocyclic lactones and LEV). A correlation between IVM-resistance and an increased frequency of β-tubulin alleles which confer BZ-resistance was reported in a large number of *H. contortus* isolates (Mottier and Prichard, 2008), suggesting that pre-existing resistance to other anthelmintic classes may contribute to the rapid development of MPTL-resistance in these populations. Currently the best defined anthelmintic resistance mechanism is that of BZs, which have been shown to bind to β-tubulin disrupting the formation of microtubules that are essential structural components of eukaryotic cells (Barrowman et al., 1984; Lacey, 1988; Prichard, 1990). Resistance to BZs has been strongly associated to three polymorphisms Phe167Tyr, Glu198Ala and Phe200Tyr in the β-tubulin isotype-1 (*Tci-tub-1*) gene in *T. circumcincta* (Kwa et al., 1994; Elard et al., 1999; Silvestre and Humbert, 2000). The development of pyrosequencing assays specifically targeting these amino acid substitutions in *T. circumcincta* has enabled genotyping to distinguish between BZ-susceptible and BZ-resistant larvae (Skuce et al., 2010).

Our aim in the present study was to determine whether the genetic basis of MPTL-resistance in *T. circumcincta* is similar to that described in laboratory-derived and field strains of MPTL-resistant *H. contortus* (Kaminsky et al., 2008; Rufener et al., 2009; Bagnall et al., 2017). For the purpose of this study, we had access to a unique resource, namely three MPTL-sensitive isolates of *T. circumcincta* and three MPTL resistant isolates which had been derived from them under defined laboratory conditions (Bartley et al., 2015), thus providing a valuable resource for phenotypic and genotypic comparisons. Generation of the full-length cDNA sequences and subsequent conceptual translation in amino acid sequence allowed comparisons of the *Tci-mptl-1* gene from these populations. Genotyping individual third-stage infective larvae (L_3_) at the *mptl-1* locus, along with ten microsatellite loci, allowed comparisons between these six experimentally derived populations, as well as three further UK field populations of *T. circumcincta*, one of which has been isolated prior to the release of Zolvix® (and thus was assumed MPTL-susceptible) and two MPTL-resistant populations which were both derived from the same field isolate but collected pre- and post-Zolvix® treatment. Pyrosequencing assays were utilised to investigate whether selection with MPTL has any interaction with the genetic mechanism also responsible for resistance to an older broad-spectrum anthelmintic family, in this case BZ. If this was the case, worms surviving MPTL treatment might be expected to show changes in the incidence of particular genetic markers (i.e. β-tubulin gene) similar to worms surviving BZ treatment.

## 2 Material and methods

### 2.1 Parasite populations

Nine populations of *T. circumcincta*, six of which had been developed and maintained in the laboratory over a number of years while the remaining three were field derived isolates, were utilised in this study:

i) MTci2: obtained from the Central Veterinary Laboratories (Weybridge, UK) in 2000, this isolate was originally sourced from the field pre-1970, prior to routine use of LEV and IVM anthelmintics. Susceptibility to all broad spectrum anthelmintics has been verified (Skuce et al., 2010; unpublished data).
ii) MTci5: originally isolated as an IVM-resistant strain from a closed flock on a Scottish lowland sheep farm in 2001 (Sargison et al., 2001), this strain has since been maintained in the laboratory and has been shown to be phenotypically resistant to BZ, LEV and IVM (Bartley et al., 2004, 2005).
iii) MTci7: an isolate originally collected from a mixed livestock farm based in central Scotland in 2005, this strain has similarly been maintained in the laboratory ever since and has been shown to be resistant to BZ, LEV, IVM and MOX (Sargison et al., 2005; Wilson and Sargison, 2007).
iv) MTci2-11: artificially selected *in vivo* from the MTci2 parental isolate after 13 rounds of sub-optimal dosing with Zolvix® and subsequently shown to be phenotypically resistant to MPTL (Bartley et al., 2015).
v) MTci5-13: derived from the MTci5 parental isolate following 9 rounds of sub-optimal dosing of the hosts with Zolvix®. Subsequently confirmed to be phenotypically resistant to MPTL (Bartley et al., 2015).
vi) MTci7-12: derived from the MTci7 parental isolate following 12 rounds of sub-optimal dosing of the hosts with Zolvix® and subsequently shown to be phenotypically resistant to MPTL (Bartley et al., 2015).

Laboratory strains above have been maintained by passage through parasite-naïve lambs without subsequent exposure to anthelmintics. A further two field isolates were included in this study.

vii) MFie18: a multi-species field isolate collected in 2017 from the same mixed livestock farm based in central Scotland from which MTci7 was isolated 10 years earlier, and has since been confirmed to be phenotypically MPTL-resistant (Hamer et al., 2018; Bartley et al., 2019). Two *T. circumcincta* populations of MFie18 were examined, one pre-Zolvix® treatment (MFie18_Pre) and another collected 5–7 days post-Zolvix® treatment (MFie18_Post).
viii) UK049L09: a multi-species field population (72.5% *T. circumcincta*) collected from lambs on an upland farm in North Scotland in 2009 (Burgess et al., 2012). This isolate is considered to be MPTL-susceptible as its isolation pre-dated the UK launch of Zolvix® in 2014.

### 2.2 Generation of full-length Tci-mptl-1 gene cDNA sequences from UK isolates

Pools of approximately 1000 L_3_ from each of the populations of *T. circumcincta* maintained at Moredun Research Institute, and 40 adult male worms of the field isolate, MFie18_Post, collected post mortem, were snap frozen in liquid nitrogen. RNA was extracted using TRIzol Reagent (ThermoFisher Scientific), following the manufacturer's instructions. Approximately 100 ng of RNA was reverse transcribed to first-strand cDNA using the SuperScript® IV Reverse Transcriptase (Invitrogen) and an oligo dT primer. Amplification was conducted using Q5® high-fidelity DNA polymerase (New England Biolabs) in a final volume of 25 μl (Q5®high-fidelity reaction buffer, 0.2 mM dNTPs, 0.5 μM of each primer, 0.02 U/μl Q5® high-fidelity DNA polymerase and 1 μl template cDNA), using touchdown PCR thermocycling conditions as follows: initial denaturing step at 98 °C for 2 min; 10 three-step cycles of 98 °C for 10 s, T_anneal_ for 20 s and 72 °C for 90 s, with decreasing annealing temperature by 0.5 °C per cycle from the (T_anneal_ + 5 °C) to the T_anneal_; this was followed by 30 three-step cycles of 98 °C for 10 s, T_anneal_ for 20 s and 72 °C for 90 s with a final extension step of 72 °C for 5 min. The *Tci-mptl-1* gene was amplified in two sections using a nested-PCR approach: 5′ section amplified with MPTL-SL1 (5′-ATTACCCAAGTTTGAGATCTCAAGG-3′) and MPTL-degR1 (5′-TTTTGCGCATAGGCYTGYTTCAT-3′), followed with semi-nested-PCR with primer pair MTPL-SL1 and MPTL-degR2 (5′-CATRAGAAGCGGCATCTCCAT-3′); the 3′ section was initially amplified with primer pair MPTL-F2 (5′-TGGTCATATACGTACAGAAGCAGG-3′) and MPTL-degR3 (5′-GGMTCTTCMATAGATTTATRAAAATAC-3′) followed by semi-nested-PCR with primer pair MPTL-F2 and MPTL-degR4 (5′-GTGCAGTCAAACGGAACACTTCG-3′). The amplicons were confirmed with gel electrophoresis and cDNA was purified from excised gel slices using the Monarch® DNA Gel Extraction Kit (New England Biolabs). The blunt-ended amplicons were ligated into pJET1.2/blunt cloning vector using the CloneJET PCR cloning kit (ThermoFisher Scientific) as per the manufacturer's instructions and were used to directly transform JM109 competent *E. coli* cells (Promega). Sequencing of the purified plasmid DNA was outsourced to Eurofins Genomics (Germany) using the pJET1.2for and pJET1.2rev sequencing primers. Sequence data were aligned and analysed using SeqMan Pro™ software from DNASTAR® Lasergene® v15 with default parameters. Contiguous sequences were generated from sequence data from a minimum of three clones for each population.

### 2.3 Preparation of genomic DNA template from single worm lysates

Thirty-one individual L_3_ were transferred to 96-well plates containing 10 μl of Direct PCR Lysis Buffer (Viagen) supplemented with 1 mg/ml proteinase K solution (Qiagen) and 0.05 M DTT (Qiagen). A negative control well, containing only supplemented lysis buffer, was included for each population. Lysis was conducted at 60 °C for 2 h followed by incubation at 85 °C for 45 min to inactivate the proteinase K. A 20-fold dilution of the neat lysate was prepared of which 1 μl was used as the gDNA template for subsequent PCR amplifications.

### 2.4 Molecular verification of T. circumcincta species

Successful worm lysis and molecular verification of larval identity was confirmed by PCR-amplifying a fragment of the nuclear ribosomal internal transcribed spacer-2 (ITS-2) region with the *T. circumcincta*-specific primers TcF (5′-ATACCGCATGGTGTGTACGG-3′) and TcR (5′- CAGGAACGTTACGACGGTAAT-3′) from Burgess et al. (2012). Each 10 μl reaction contained GoTaq® flexi buffer, 2.5 mM MgCl_2_, 0.2 mM each dNTP, 0.5 μM of each primer, 0.25 U GoTaq® G2 Flexi DNA polymerase (Promega) and 1 μl of diluted gDNA lysate. Touchdown PCR was conducted with the following thermocycling conditions: 95 °C for 8 min, followed by 12 three-step cycles of 94 °C for 15 s, 62 °C for 15s reducing by 0.5 °C/cycle and 72 °C for 30 s, 25 three-step cycles of 94 °C for 15 s, 56 °C for 55 s and 72 °C for 30 s, followed by a final extension step at 72 °C for 7 min.

### 2.5 PCR amplification of microsatellite markers

Genotyping of 30–31 individual L_3_ from each of the nine populations was conducted using a panel of 10 previously described microsatellite loci as population markers for *T. circumcincta*: MTG15, MTG67, MTG68, MTG73 (Grillo et al., 2006), Tc2066, Tc2467, Tc4504, Tc7989, Tc13604 and Tc22274 (Redman et al., 2015). The primers used in PCR amplification and their corresponding annealing temperatures are listed in Table 1. Fluorescent labelling of the 5′ end of sense primers with FAM or HEX dyes facilitated DNA fragment size analysis by capillary electrophoresis (outsourced to MRC PPU DNA Sequencing and Services, University of Dundee) allowing multiplexing based on their expected sizes and labelling. PCR was performed in 20 μl reactions containing 45 mM Tris-HCl (pH 8.8), 11 mM (NH_4_)SO_4_, 4.5 mM MgCl_2_, 6.7 mM 2-mercaptethanol, 4.4 μM EDTA, 113 μg/ml BSA, 2% Tween, 1 mM each dNTP, 0.5 μM of each primer, 0.5 U GoTaq® G2 Flexi DNA polymerase (Promega) and 1 μl of diluted gDNA lysate. The PCR thermocycling conditions were: 94 °C for 2 min, followed by 40 three-step cycles of 94 °C for 15 s, specific annealing temperature for 55 s and 72 °C for 30 s, followed by a final extension step at 72 °C for 15 min. To determine the genotype of each sample the individual electropherograms were analysed in the Geneious ver 10.2.5 (Biomatters Ltd) software package. Using ROX500 as the internal size standard, peaks were allocated into bins based on the repeat unit length and expected size range of the amplicons (Table 1).

**Table 1:** Panel of 10 microsatellites used in the genetic analysis of *T. circumcincta* populations. The optimal annealing temperature (*T*_*A*_) for each primer pair is listed along with the size range of the amplified alleles. Primer sequences are those reported by^a^Grillo et al. (2006) and^b^Redman et al. (2015).

Microsatellite	Repeat Sequence	*T*_*A*_ (°C)	Size range of alleles (bp)	Primer sequences (5′ → 3′) (Sense (S) and Antisense (A))
MTG 15^a^	(GT)_6_GC (GT)_6_GGGTTTGT	58	233–275	S: TGCAAGGAAACTGCTAAGAAGGAG A: ATCATGGAACCTTGATACCGCAAG
MTG67^a^	(CT)_14_	58	172–192	S: CAAGTCGTTTAGGCACGTCTGG A: CAGGGCGGAACCCAATTGATCG
MTG68^a^	(ACA)_2_ (ACC)_2_TCG (ACA)_4_ACTACAACCA CAACTACG (ACAACC)_2_	50	420–453	S: ATCACCAGGCGGCTGCTACG A: CGAAAAGTAGAGTATGAGC
MTG73^a^	(TGC)_2_TGTTGC (TGT)_2_ (TGA)_3_TG (TGA)_2_T AA (TGA)_2_AAATA (TGA)_2_	45	148–157	S: CCTTGTATAAATTCGAAGC A: GTAGTAGTGATTAACTTCCG
Tc 2066^b^	[GGCGAGTA]_9_GGCGATTAGGCGAGAA GGCGTGTAGGTGAGTAGGCGAG	50	189–309	S: GAGCAACGACTGAACCTCAC A: GCTGGAAGCATATTCTGCGC
Tc2467^b^	[TTTA]_15_TCTATT	54	149–224	S: AACGCTTTGAACCGTGTCGG A: GCTGCCACATCAGCTTAGA
Tc4504^b^	[ACAT]_13_ACAGATACAGACA	50	228–276	S: TTATCACACCACTTCATT A: GTCTTTAAACGCTAAATA
Tc7989^b^	[GTCT]_17_GTCCGTTTGTGTG	50	133–232	S: GATCTCACGTACTATGAA A: CTATTGAATGTCGTACAG
Tc13604^b^	[CAGGTA]_12_TAGGTACAGGCACAG	50	250–386	S: CGATAAATGGTATTATCTG A: GCTGCTATTAGAGGATAT
Tc22274^b^	[TGTA]_17_TGTCTGAATGT	54	189–329	S: ACAAAGTGCTCAAGTTAG A: GGGGGTTCTATATACAGTA

Statistical analyses to estimate the expected heterozygosity (*H*_e_) was based on Nei's measure of genetic distance (Nei, 1978), unbiased for sample size. Observed heterozygosity (*H*_o_), inbreeding coefficient (*F*_IS_) and Exact tests for Hardy-Weinberg equilibrium (HWE) (1,000,000 steps in Markov chain and 100,000 dememorisation steps) were calculated in Arlequin ver 3.5.2.2 (Excoffier and Lischer, 2010). Sequential Bonferroni correction was used to correct for multiple tests, significant departure from HWE had *P*-values <0.005. Population pairwise *F*_ST_ values were (16,002 permutations for significance; Mantel test, 1000 permutations) and analysis of molecular variance (AMOVA) was used to determine population differentiation. FreeNA (Chapuis and Estoup, 2007) was utilised to estimate the number of alleles per locus per population and the null allele frequencies. Principle coordinates analyses (PCoA) were conducted in GenAlEx ver 6.501 (Peakall and Smouse, 2006, Peakall and Smouse, 2012).

### 2.6 PCR amplification of exons 7 and 8 of Tci-mptl-1 from individual larvae

Diluted larval gDNA lysates from the nine populations were used as template DNA to PCR amplify an amplicon spanning exons 7 and 8 of the *Tci-mptl-1* gene including the intervening intron, from 30 to 31 individual larvae from the nine *T. circumcincta* populations. Degenerate primers MPTL-EXSP-F (5′-TCGGYAGTTGGACGTTTG-3′) and MPTL-EXSP-R (5′-CTGAAGAAYCCAACAATTGAGATC-3′) amplified a 547 bp fragment of *Tci-mptl-1*. Each 20 μl reaction contained 1X Q5® reaction buffer, 0.2 mM each dNTP, 0.5 μM of each primer and 0.4 U Q5® High-Fidelity DNA Polymerase (New England Biolabs). Touchdown PCR was conducted with the following thermocycling conditions: 98 °C for 2 min, followed by 10 three-step cycles of 98 °C for 10 s, 67 °C for 20 s reducing by 0.5 °C/cycle and 72 °C for 60 s, 30 three-step cycles of 98 °C for 10 s, 62 °C for 20 s and 72 °C for 60 s, followed by a final extension step at 72 °C for 5 min. Purification of the amplicons and DNA Sequencing was outsourced to Eurofins Genomics (Germany) and sequencing data was generated in both the sense and antisense directions using the MPTL-EXSP-F and MPTL-EXSP-R primers as sequencing primers. Sequencing data were analysed using default parameters in SeqMan Pro™ software from DNASTAR® Lasergene® v15. The location of exon-intron boundaries was identified using the Splign program (Kapustin et al., 2008). The nucleotides at three polymorphic bases of exon 7 (bases 69, 126 and 132) were recorded along with fifteen polymorphic sites in exon 8 (bases 6, 29, 35, 38, 41, 50, 51, 63, 65, 71, 92, 101, 104, 116 and 128), highlighted in Supplementary Fig. S5. Individuals with missing data at any of the polymorphic bases were excluded. Each different combination of polymorphisms was assigned a genotype number.

### 2.7 Pyrosequencing

The effect of selecting MPTL-resistant strains from MPTL-susceptible parental isolates on β-tubulin, a gene not thought to be targeted by MPTL, was investigated using pyrosequencing assays to examine the prevalence of three mutations in the *Tci-tub-1* gene. Two regions of *Tci-tub-1* gene were PCR amplified from diluted gDNA lysates of individual larvae from the nine populations of *T. circumcincta* to provide gDNA template for pyrosequencing assays (Skuce et al., 2010), using PyroMark™ (Qiagen) following the manufacturer's protocol. Amplification of the first region, that included codon Phe167Tyr of β-tubulin (Silvestre and Cabaret, 2002), was conducted with 50 μl reactions containing 1X NovaTaq™ Hot Start Mastermix, 0.4 mM Tci167for primer (5′-GCATTCTTTGGGAGGAGGTA-3′), 0.185 mM biotinylated Tci167rev-Bio primer (5′- TGCACCTCGAGAACCTGTACATA-3′), and 1.5 mM MgCl_2_ and 4 μl template gDNA. The second region targeted the polymorphic codons Glu198Ala (Ghisi et al., 2007) and Phe200Tyr (Kwa et al., 1994) of the β-tubulin isotype-1 gene. Each 50 μl PCR reaction 1X NovaTaq™ Hot Start Mastermix, 0.185 mM Tci200for-Bio biotinylated primer (5′- ACCTTACAATGCCACTCTTTCTG-3′), 0.4 mM Tci200rev (5′- GCGGAAGCAGATATCGTACAG-3′), 4.5 mM MgCl_2_ and 4 μl of template gDNA. The PCR thermocycling conditions for both *Tci-tub-1* fragments were: 94 °C for 10 min, followed by 40 three-step cycles of denaturing at 94 °C for 30 s, primer annealing at 58 °C for 30 s and an extension phase of 72 °C for 30 s, with a final extension step at 72 °C for 5 min. The amplicons were purified by conjugating the biotin-labelled PCR products to streptavidin-coated sepharose beads, and then added to a PyroMark™ Q96 Plate Low (Qiagen) containing 38.4 μl PyroMark™ Annealing Buffer (Qiagen), 1.6 μl PyroMark™ Sequencing Buffer (Qiagen) and 0.4 μM sequencing primer (Tci167Seq (5′-CGGATAGAATCATGGCT-3′) or Tci200Seq (5′- RGAGCYTCATTATCGATR-3′)), then incubated at 80 °C for 2 min to anneal the sequencing primer to the PCR amplicon. *De novo* sequencing of the plate was conducted using a PyroMark ID instrument (Biotage) in SNP identification mode.

## 3 Results

### 3.1 Generation of full-length sequences of Tci-mptl-1 gene from UK isolates of T. circumcincta

**Fig. 1:**
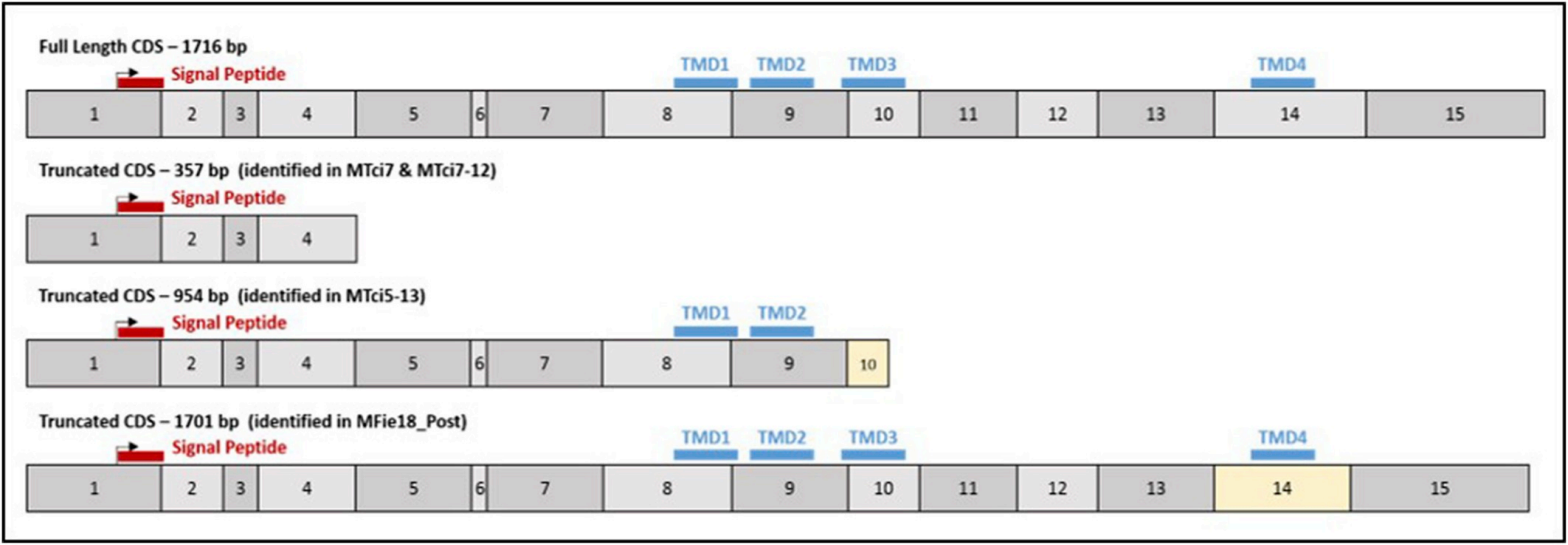
Schematic showing the full-length and truncated versions of the *Tci-mptl-1* gene identified in different populations of *T. circumcincta*. Exons are represented by numbered boxes and start codons by arrows. Mis-spliced exons are shown in yellow, the locations of the signal peptide (red) and four predicted transmembrane domains (blue) are also shown.

Full-length cDNA sequences (1716 bp) for *Tci-mptl-1*, a candidate gene for MPTL-resistance, were generated from seven of the nine populations of *T. circumcincta* (not UK049L09 or MFie18_Pre) and aligned (Supplementary Fig. S1), then conceptually translated into amino acid (AA) sequences (571 AA) (Supplementary Fig. S2). Each full-length consensus coding sequence (CDS) was derived from a minimum coverage of three separate sequencing runs (GenBank accession numbers listed in Table 2). Alignments of the sequence data were conducted using the online multiple sequence alignment tool, Clustal Omega (accessed at: www.ebi.ac.uk/Tools/msa/clustalo/). The percentage identity between *Tci-mptl-1* from each of the populations was calculated (Supplementary Table S3). Sequencing of the *Tci-mptl-1* gene was not exhaustive, therefore, the sequence data generated were assumed to represent the most abundant versions of the gene in each respective pool of larvae. Comparisons of the full-length (1716 bp) consensus CDS from the seven populations of *T. circumcincta* shared 95.4–98.6% identity. Truncated forms of the *Tci-mptl-1* gene were also identified in the MTci7 (357 bp), MTci7-12 (357 bp), MTci5-13 (954 bp) and MFie18_Post (1701 bp) populations of *T. circumcincta*, summarised in Fig. 1. The truncated isoforms of *Tci-mptl-1* from these populations showed a similar level of homology along the length of the truncated gene product when compared to their full-length form of the *Tci-mptl-1* gene (between 92.2 and 99.7% identity). A transversion mutation (T to A) at base 356 was observed in both the MPTL-susceptible MTci7 and the MTci7-12 MPTL-resistant populations. This resulted in the loss of transmembrane domains (TMD) 1–4 by introducing a premature stop codon truncating the *Tci-mplt-1* gene. In the MFie18_Post population, an in-frame deletion of 15 bases in exon 14 shortened the *Tci-mptl-1* gene by 5 amino acids but did not alter the sequence in TMD4. An insertion of 29 bp in exon 10 of the *Tci-mptl-1* gene of MTci5-13 introduces a premature stop codon resulting in the loss of exons 10 to 15 and the subsequent loss of TMD3 and TMD4. This insertion was not observed in the MTci5 population from which the MTci5-13 population was derived through sub-dosing with Zolvix®. The predicted full-length amino acid sequences generated from the seven populations of *T. circumcincta* shared >98% identity. Amino acid substitutions were observed at three positions in the *Tci-mptl-1* gene: D18E (MTci5-13), E94D (MTci5-13) and F569L (MTci5, MTci7 MTci2-11 and MTci5-13). The amino acid substitutions identified were not found exclusively in either the MPTL-susceptible or the MPTL-resistant populations of *T. circumcincta*.

**Table 2:** Summary of mutations occurring in the *Tci-mptl-1* gene in seven different populations of *T. circumcincta*.

*T. circumcincta* Population	*MPTL Resistance Status*	*Tci-mptl-1* Product (bp)	Summary of Mutation	GenBank Accession Number
MTci2	Susceptible	1716	Full-length CDS	MK871751
MTci5	Susceptible	1716	Full-length CDS	MK871752
MTci7	Susceptible	1716	Full-length CDS	MK871753
MTci7_Alt	Susceptible	357	Transversion in exon 5 from T to A that leads to the introduction of a premature stop codon which truncates the gene leading to loss of all 4 transmembrane domains and regions conserved between nAChR α subunits	MK871754
MTci2-11	Resistant	1716	Full-length CDS	MK871755
MTci7-12	Resistant	1716	Full-length CDS	MK871756
MTci7-12_Alt	Resistant	357	Transversion in exon 5 from T to A that leads to the introduction of a premature stop codon which truncates the gene leading to loss of all 4 transmembrane domains and regions conserved between nAChR α subunits	MK871757
MTci5-13	Resistant	1716	Full-length CDS	MK871758
MTci5-13_Alt	Resistant	954	Insertion of 29 bp in exon 10 introduces a premature stop codon leading to the loss of exons 10 to 15 and transmembrane domain 4	MK871759
MFie18_Post	Resistant	1716	Full-length CDS	MK871760
MFie18-Post_Alt	Resistant	1701	In-frame deletion of 15 bp leading to a truncated gene	MK871761

### 3.2 Microsatellite marker analyses

Microsatellite analysis was used to investigate the genetic structure of the nine populations of *T. circumcincta* included in this study. Larvae for which a microsatellite marker repeatedly failed to amplify were considered to be null homozygotes at that locus. The total number of microsatellite alleles varied between two (MTG73) and 23 (Tc7989) confirming that all of the markers used were indeed polymorphic. The field isolate, UK049L09, appeared to be the most polymorphic with a mean number of alleles at each locus of 12.2, whereas the MTci2-11 strain was least polymorphic with a mean number of alleles at each locus of 7.9. The mean allele richness was 10 and the expected heterozygosity (*H*_e_) of the 9 populations studied was 0.789 (range: 0.721–0.842) which is consistent with that reported by Redman et al. (2015).

**Fig. 2:**
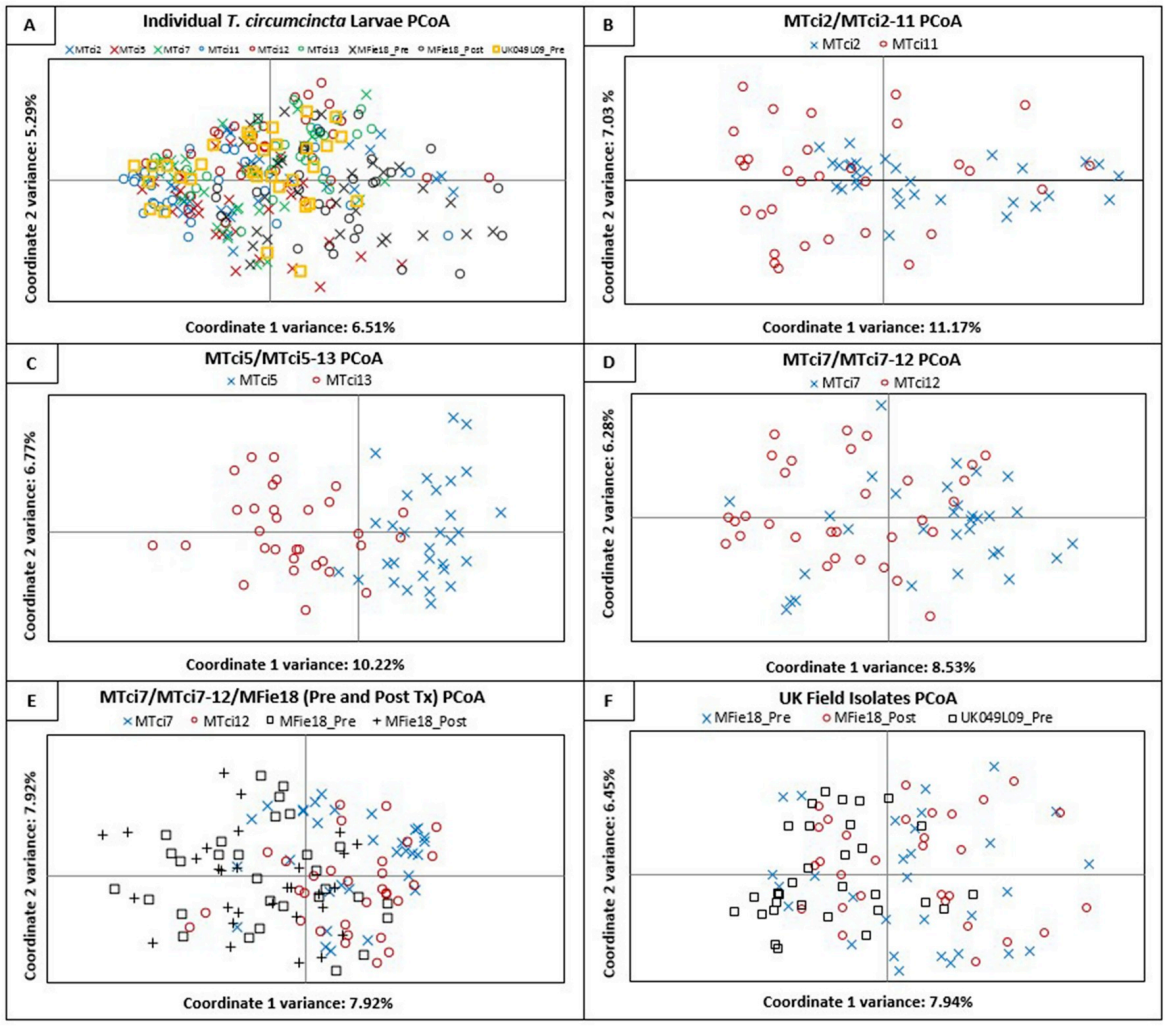
Principle coordinates analysis was performed using GenAlEx ver 6.501 (Peakall & Smouse, 2006, 2012) on multilocus genotypes at ten loci from 30 to 31 individual larvae belonging to nine populations of *T. circumcincta*. Panel A shows a comparison of genotypes of all individuals from each population at ten microsatellite loci. Panels B–D show comparisons between MPTL-susceptible populations and the MPTL-resistant strains selected from these populations. Comparisons between a MPTL-susceptible population, MTci7, isolated from a Scottish farm in 2007 and MTci7-12 experimentally derived from MTci7 and the related field-selected (MFie18) MPTL-resistant populations are shown in Panel E. Panel F shows a comparisons between a MPTL-resistant UK-field isolate, MFie18 pre- and post-Zolvix® treatment, and a MPTL-susceptible UK field isolate collected in 2009, prior to the release of Zolvix® in the UK. The x- and y-axis of the graph show the percentage variation explained by the first and second coordinates, respectively.

AMOVA (Supplementary Table S4) highlighted that the majority of the variation was distributed within populations (96.49%) rather than between isolates (3.51%; variance: 0.068; *P* < 0.01). The extent of population sub-structuring between isolates was quantified by calculating the *F*_ST_ values, based on the ten polymorphic loci, in pairwise comparisons between the nine populations. The pairwise comparisons confirmed a very low level of between-population genetic differentiation (*F*_ST_ values between 0.031 and 0.076) between the MPTL-susceptible isolates (MTci2, MTci5 and MTci7) and their respective MPTL-resistant descendants (MTci2-11, MTci5-13 and MTci7-12). The Global *F*_ST_ value of 0.038 (mean over all populations, 10,100 permutations) is low, possibly due to the isolates of *T. circumcincta* sharing a similar geographical origin (all UK isolates). To plot the genetic distance between individual larvae from each of the nine populations, PCoA was conducted (Fig. 2, Panel A), coordinate 1 and 2 represent 6.51% and 5.29% of the variation supporting the pairwise *F*_ST_ values (Table 3). Pairwise comparison between the initial MPTL-susceptible population and the descendent MPTL-resistant population selected by Zolvix® treatment showed that the MTci2/MTci2-11 and the MTci7/MTci7-12 populations had not undergone a great deal of genetic divergence. The comparison between MTci5 and MTci5-13 showed the greatest genetic divergence of the three population pairs as it partitions into two distinct clusters (Panel C, Fig. 2). Comparisons of the MTci7, MTci7-12 and MFie18 populations showed little divergence (Panel E, Fig. 2), which might be expected as MTci7 and MFie18 were isolated from the same farm 10 years apart. In addition, these data also highlights that differences in MTci7-12 population are unlikely to be artefacts of the MPTL-resistance selection process due to their similarity to the MPTL-selected field isolate.

**Table 3:** Population pairwise *F*_ST_ values for nine populations of *T. circumcincta* based on ten microsatellite markers (number of permutations = 16,002) calculated in Arlequin (ver 3.5.2.2). Higher incidence of nucleotide substitutions that occur within rather than between populations is indicated by negative values.

Population	MTci2	MTci5	MTci7	MTci11	MTci12	MTci13	MFie18 Pre	MFie18 Post
MTci5								
*F*_ST_	0.0031							
*P-value*	0.3510							
*(s.d.)*	(0.0039)							
MTci7								
*F*_ST_	−0.0180	0.0391						
*P-value*	0.9908	<0.001						
*(s.d.)*	(0.0007)	(0.0000)						
MTci11								
*F*_ST_	0.0312	0.0478	0.0682					
*P-value*	0.0028	<0.001	<0.001					
*(s.d.)*	(0.0004)	(0.0000)	(0.0000)					
MTci12								
*F*_ST_	0.0143	0.0425	0.0404	0.1012				
*P-value*	0.0244	<0.001	<0.001	<0.001				
*(s.d.)*	(0.0012)	(0.0001)	(0.0000)	(0.0000)				
MTci13								
*F*_ST_	0.0316	0.0763	0.0662	0.1345	0.0727			
*P-value*	<0.001	<0.001	<0.001	<0.001	<0.001			
*(s.d.)*	(0.0001)	(0.0000)	(0.0000)	(0.0000)	(0.0000)			
MFie18_Pre								
*F*_ST_	−0.005	0.0453	0.0058	0.0885	0.0096	0.0471		
*P-value*	0.7872	<0.001	0.3523	<0.001	0.1724	<0.001		
*(s.d.)*	(0.0030)	(0.0000)	(0.0037)	(0.0000)	(0.0029)	(0.0000)		
MFie18_Post								
*F*_ST_	0.0310	0.0464	0.0275	0.1137	−0.0014	0.0459	−0.0007	
*P-value*	0.0046	<0.001	0.0040	<0.001	0.7018	<0.001	0.73,799	
*(s.d.)*	(0.0005)	(0.0001)	(0.0005)	(0.0000)	(0.0035)	(0.0000)	(0.0036)	
UK049L09_Pre								
*F*_ST_	−0.0049	0.0010	0.02403	0.0690	0.0108	0.0451	−0.0300	−0.0278
*P-value*	0.8244	0.5572	<0.001	<0.001	0.0568	<0.001	0.9998	0.9981
*(s.d.)*	(0.0028)	(0.0039)	(0.0001)	(0.0000)	(0.0022)	(0.0000)	(0.0001)	(0.0003)

### 3.3 Genotyping using polymorphisms present in exons 7 and 8 of Tci-mptl-1 gene

**Fig. 3:**
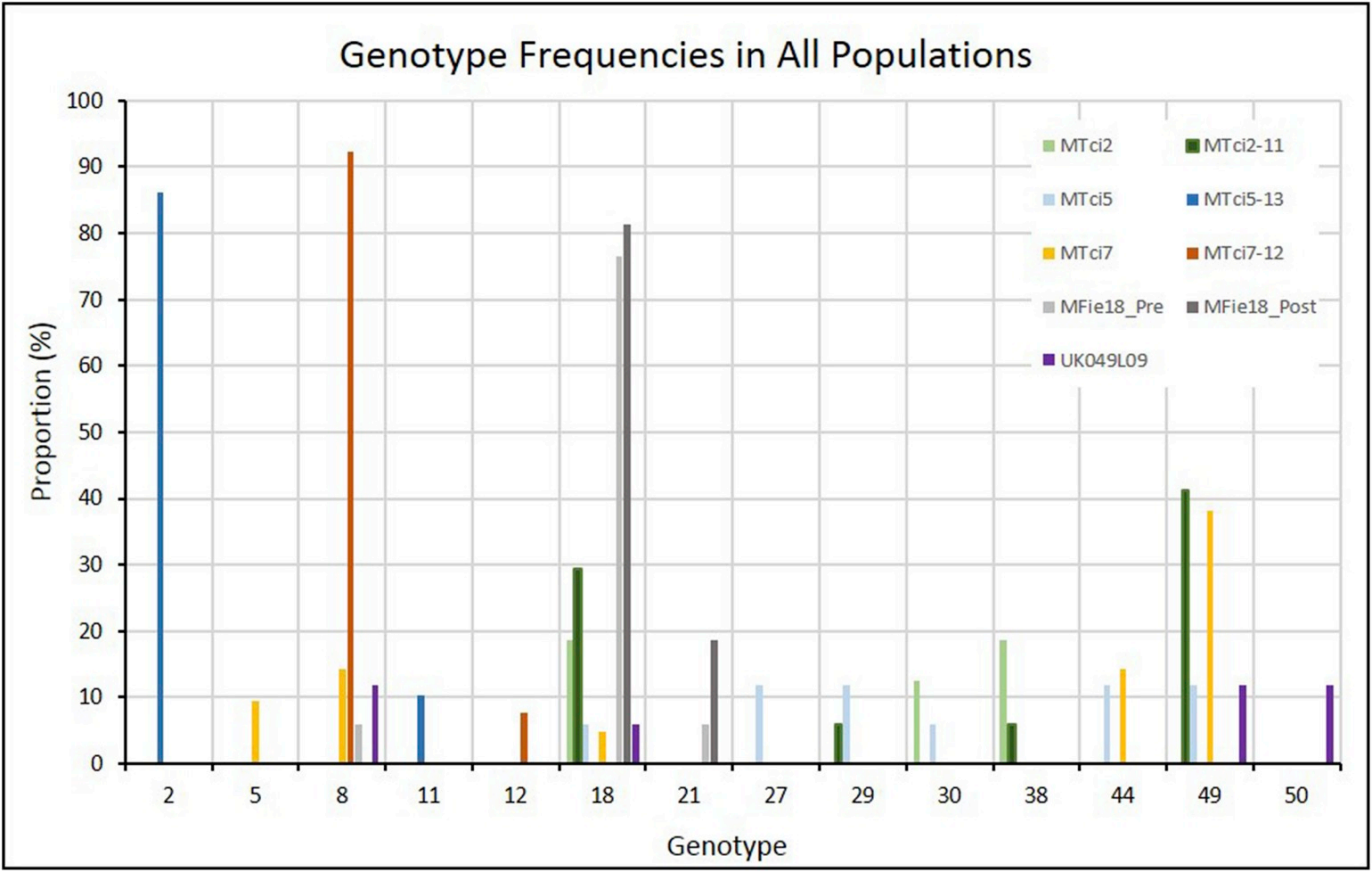
Assigned genotype numbers based on combinations of polymorphisms present in exons 7 and 8 of *Tci-mptl-1*. The bar chart shows the proportion of larvae from each population that possess each particular genotype. Unique alleles (i.e. those that are present only in a single larva) were removed to highlight the genotypes that occurred more than once in the larvae from the nine populations of *T. circumcincta*.

**Fig. 4:**
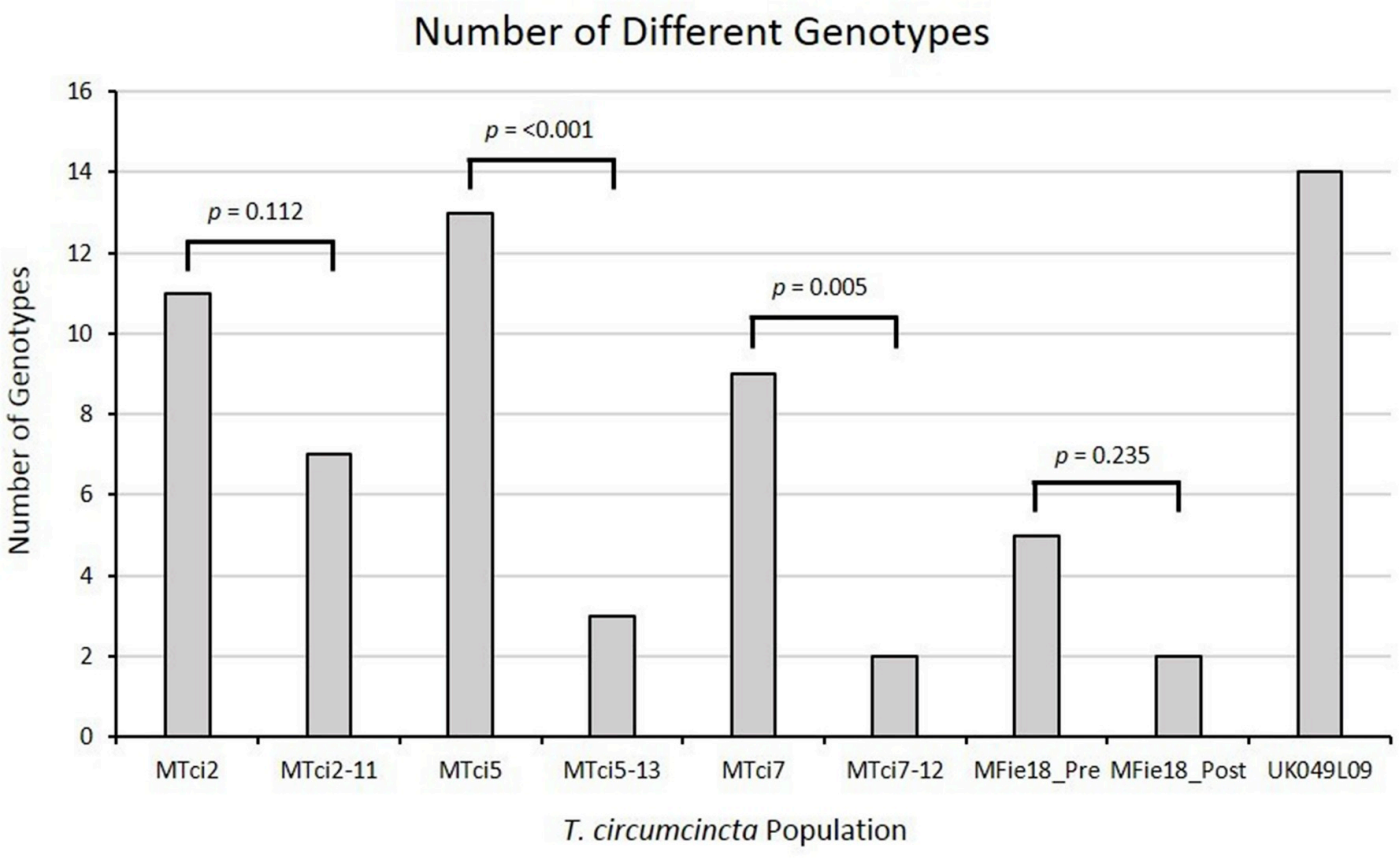
The number of different genotypes at exons 7 and 8 of *Tci-mptl-1*, was compared in MPTL-susceptible populations and MPTL-resistant populations. Chi-squared tests were conducted to highlight any significant differences (*p < 0.05*) between related populations of *T. circumcincta*.

Sequencing results for *Tci-mptl-1* highlighted a polymorphic region encompassing exon 7, intron 8 and exon 8. The amplification and sequencing of this 547 bp gDNA fragment of the *Tci-mptl-1* gene, across all the individuals studied, allowed the allocation of genotypes based on the combinations of selected polymorphisms. Fifty-one distinct genotypes were identified with particular genotypes more common than others; of the 51 genotypes identified only 14 occurred in two or more larvae. These are plotted in Fig. 3. Comparisons between the MPTL-susceptible parental isolate MTci7 and its MPTL-resistant derived progeny, MTci7-12, showed that the frequency of genotype 8 increased from 14.3% to 92.3% respectively. Furthermore genotype 2 was not identified at all in individuals of the MTci5 isolate but represented 86.2% of the genotypes in the MTci5-13 strain. In contrast, the MPTL-resistant field isolate, MFie18, had a high abundance of genotype 18 both pre- and post-monepantel treatment suggesting that genotype 18 did not undergo further purifying selection in this isolate (possibly because selection had occurred prior to its isolation). Genotypes 2, 8, 18 and 49 occurred most often in these populations, accounting for 111 of 176 larvae analysed. Comparisons of the number of different genotypes in the parental isolates relative to their respective selected MPTL-resistant strains (Fig. 4) suggested that there had been a reduction in the number of genotypes present in the resistant populations compared to the MPTL-susceptible population from which they were derived in all cases. However these apparent reductions were statistically significant (*P* < 0.05) only in the case of MTci5-13 and MTci7-12 MPTL-resistant populations. Comparison of post-Zolvix® treatment field isolate, MFie18_Post, with the same isolate pre-Zolvix® treatment (MFie18_Pre), similarly appeared to show a reduction in the number of genotypes although again this was not statistically significant (possibly because selection had occurred prior to its isolation).

The results support the hypothesis that MPTL treatment is applying purifying selection pressure on the *Tci-mptl-1* gene with a reduction in the total number of genotypes present within a population, as well as an increase in incidence of specific genotypes.

### 3.4 Pyrosequencing of mutations in the beta-tubulin isotype 1 gene

**Fig. 5:**
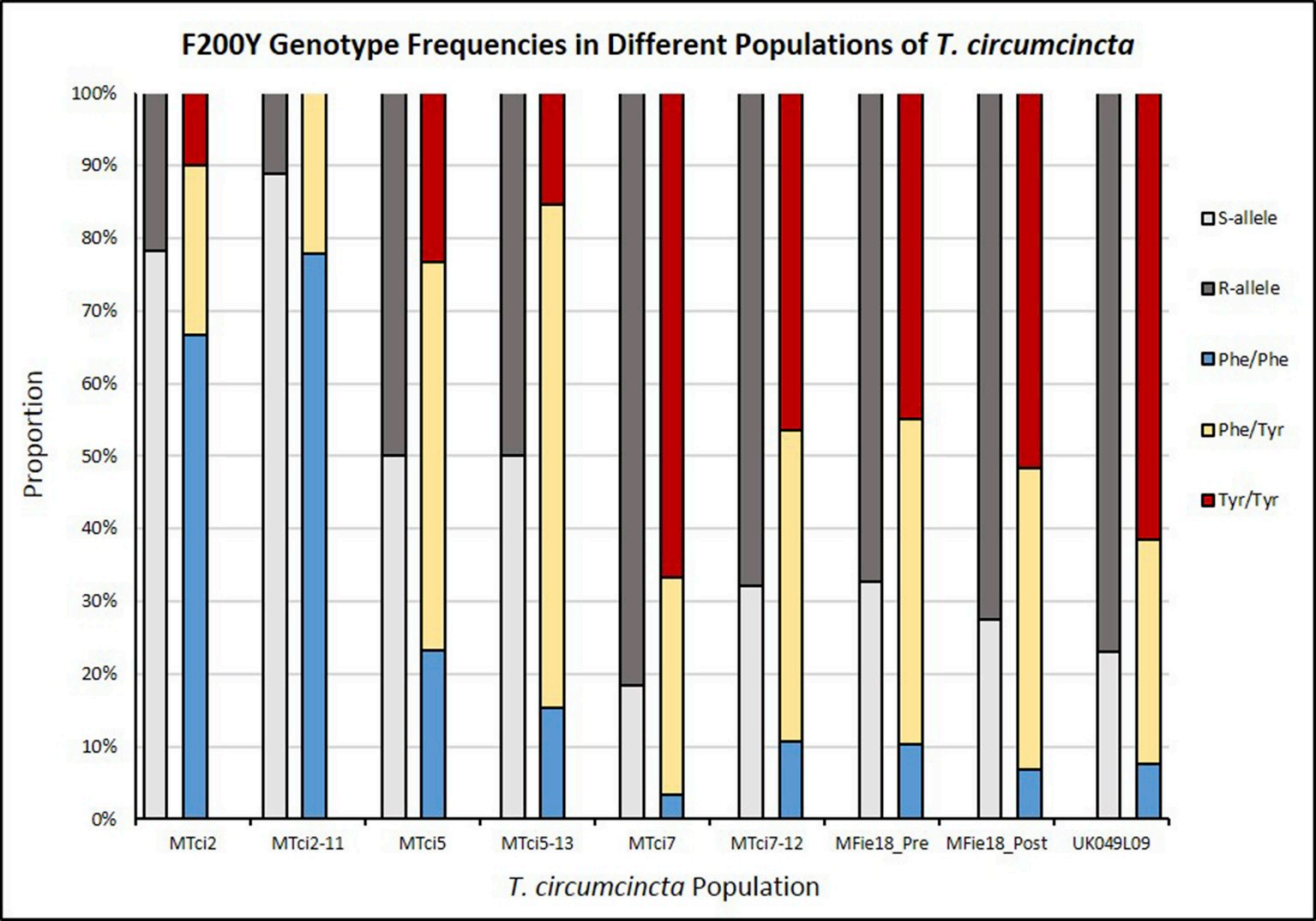
Pyrosequencing at codon 200 of *Tci-tub-1* was conducted to determine the genotypes present in individual larvae from nine different populations of *T. circumcincta*. The proportion of BZ-susceptible and BZ-resistant alleles present in each population is shown in light grey and dark grey, respectively. The proportions of genotypes present are also shown: homozygous BZ-susceptible (Phe/Phe) in blue, heterozygous BZ-resistant (Phe/Tyr) in yellow and homozygous BZ-resistant (Tyr/Tyr) in red.

Genotyping at codons 167, 198 and 200 of *Tci-tub-1* highlighted heterozygosity and homozygosity in individuals from each of the populations of *T. circumcincta* (Supplementary Table S6). One of the 265 screened larva, from the MTci5 population, was heterozygous at codon 167 (Phe/Tyr), whereas the others were homozygous Phe/Phe which confers susceptibility to BZs. All of the larvae from the nine different populations were homozygous BZ-susceptible at codon 198 (Glu/Glu). Comparisons between the MPTL-susceptible isolates and their experimentally derived MPTL-resistant populations (Fig. 5) showed a reduction in the number of homozygous BZ-resistant genotypes at position 200 (range 7.9–20.3% reductions). Comparisons of MTci2 and MTci2-11 at codon 200 revealed a 10% loss of the homozygous BZ-resistant (Tyr/Tyr) genotype and an 11% gain of homozygous BZ-susceptible (Phe/Phe) genotype, with little change in the proportion of heterozygous (Phe/Tyr) individuals. Similarly, comparisons of MTci7/MTci7-12 and MTci7/MFie18_Pre both showed increases of 7% in homozygous BZ-susceptible genotypes and decreases in the proportion of BZ-resistant homozygotes by 20.3% and 21.9%, respectively, while the proportion of heterozygous individuals increased by 12.9% and 14.8% in the respective comparisons. Comparing MTci5 and MTci5-13 showed a 7.9% decrease in homozygosity in both the BZ-susceptible (Phe/Phe) and BZ-resistant (Tyr/Tyr) genotypes and a 15.9% increase in heterozygosity at codon 200 of *Tci-tub-1*.

## 4 Discussion

This paper presents the genetic characterisation of two field isolates and three laboratory isolates of *T. circumcincta*, from which, a further three MPTL-resistant populations were derived. Studies in *H. contortus* have identified a number of loss-of-function mutations in the *Hco-mptl-1* gene that resulted in mis-spliced transcripts and premature stop codons in both laboratory-selected resistant isolates (Rufener et al., 2009) and in field isolates (Bagnall et al., 2017). Truncated versions of the MPTL-1 gene are likely to be targeted to the lysosome and degraded. None of the mutations observed in the *T. circumcincta* populations were reported in the *H. contortus* homologue although similar loss-of-function mutations resulting in the truncated forms of *Tci-mptl-1* observed in the MTci7 and MTci7-12 populations which in turn lead to the loss of transmembrane domains, will likely impede channel formation, rendering it inactive. Mutations in *Tci-mptl-1* are tolerated by *T. circumcincta* and could be expected to be rapidly selected for under drug exposure, supporting the theory that the *Tci-mptl-1* gene may be responsible for the loss of sensitivity to MPTL in these parasite populations.

Genetic characterisation was conducted at 10 neutral microsatellite loci, and at the *Tci-mptl-1* and *Tci-tub-1* loci that have been linked to MPTL and BZ resistance, respectively. Microsatellite markers were used to study the effects of monepantel treatments on the genetic structure of the *T. circumcincta* populations at ‘neutral’ loci that are not targeted by MPTL. Microsatellites are commonly used as population genetic markers in a wide range of organisms and consist of short tandem repeats (Schlötterer and Pemberton, 1998). Previous studies in nematodes have shown that microsatellite markers are often conserved between closely related populations (Grillo et al., 2006, 2008; Redman et al., 2008). The low level genetic diversity (global *F*_ST_ = 0.038) between the nine populations of *T. circumcincta* allowed genetic comparisons of genes that were under selection following exposure to MPTL-treatment. The MPTL-resistant populations and MPTL-susceptible isolates from which they were experimentally selected showed little genetic divergence, most likely due to the relatively low number of generations between them: MTci2-11 was selected from MTci2 in thirteen rounds, MTci7-12 was selected from MTci7 in twelve rounds and MTci5-13 was selected from MTci5 in nine rounds of sub-optimal dosing with Zolvix® (Bartley et al., 2015). A low level of genetic divergence may arise from the serial passaging of these *T. circumcincta* populations through parasite-free lambs and during selection with MPTL. The mean observed heterozygosity (*H*_*o*_) was significantly less than the mean expected heterozygosity (*H*_*e*_) in all populations, suggesting the presence of null alleles for some of the combinations of marker and population as seen in previous studies (Grillo et al., 2007; Hunt et al., 2008; Redman et al., 2008, 2015; Silvestre et al., 2009).

Having shown the low levels of genetic diversity at neutral loci, attention was focussed upon polymorphisms in *Tci-mptl-1*, a candidate gene involved in MPTL-resistance. A fragment of the *Tci-mptl-1* spanning exons 7 and 8, as well as the intervening intron, was amplified and sequenced from individuals from seven populations of *T. circumcincta*. A genotype number was allocated based on pattern of polymorphisms at 18 positions in exons 7 and 8 of the *Tci-mptl-1* gene. In total, fifty-one distinct genotypes were identified, with populations possessing up to fourteen different variants. This may reflect the known diversity of the *T. circumcincta* genome, given the average number of alleles at the neutral microsatellite loci per population was 10 (range: 7.9–12.2). The MFie18 field isolate has substantially fewer genotypes than the unrelated UK049L09 field isolate which was collected prior to the release of Zolvix®. The evidence presented in this study suggests that after exposure to monepantel, some genotypes/isoforms of the *Tci-mptl-1* gene that were not advantageous under exposure to MPTL were eliminated as a result of purifying selection. A similar purifying selection of advantageous sequence variants was highlighted in comparisons of a P-glycoprotein implicated in IVM-resistance (*Tci-pgp-9*) in the MTci2 and MTci5 isolates of *T. circumcincta* (Turnbull et al., 2018).

The potential impact of artificially selecting for MPTL-resistance with sub-optimal dosing, as well as selection for MPTL-resistance in the field, on the β-tubulin genotype of *T. circumcincta* was explored. Previously, a correlation between the selection for IVM-resistance and an increased frequency of β-tubulin alleles containing codons which confer BZ-resistance was reported in a large number of *H. contortus* isolates (Mottier and Prichard, 2008) and in *Onchocerca volvulus* (Osei-Atweneboana et al., 2012). Silvestre and Cabaret (2002) report that a homozygous resistant genotype (Tyr/Tyr) at either codon 167 or 200 was sufficient to confer BZ-resistance in French *T. circumcincta* populations. Ghisi et al. (2007) associated a further mutation of glutamate to alanine at codon 198 of β-tubulin isotype 1 with BZ-resistance in South African and Australian populations of *H. contortus*. The Phe167Tyr mutation in β-tubulin isotype 1 appears to be a rare mechanism of BZ-resistance in the UK populations of *T. circumcincta* as it was only observed in a single heterozygous larva of the 265 included in the present study. The Glu198Ala mutation was not observed in these nine UK populations of *T. circumcincta*. The Glu198Ala and Glu198Leu mutations have been observed in South African and Australian isolates of *H. contortus* (Ghisi et al., 2007) and in New Zealand isolates of *T. circumcincta* (Choi et al., 2017), respectively, suggesting that the mutation had arisen independently in these areas. Given the high frequency of the Phe200Tyr mutation in the UK populations, the presence of a double mutation is expected to be exceedingly rare due to the infrequency of mutation events and the close proximity of the mutations, which effectively rules out recombination between them. Additionally, the fitness cost of possessing two homozygous alterations in the β-tubulin gene might be explained by ‘mutual exclusion’ in nature where both mutations result in worm lethality (Ghisi et al., 2007; Kotze et al., 2012; Zhang et al., 2016). In general, the MPTL-selected populations derived experimentally (MTci2-11, MTci7-12, and MTci5-13) or selected in the field (MFie18) showed decreased homozygous BZ-resistant genotypes and an increase in heterozygous genotypes at codon 200 of the β-tubulin gene. The increased heterozygosity in the MPTL-resistant populations may be explained by a counter-selection of BZ-resistant homozygotes during the selection for MPTL-resistance. As the β-tubulin isotype-1 gene (Chromosome I) is remote from the DEG-3 cluster (Chromosome II) it is unlikely that genetic hitchhiking would explain these results. Reversion towards anthelmintic susceptibility in *T. circumcincta* has been modelled (Leathwick et al., 2013), and shown in response to an integrated resistance management strategies in the field (Leathwick et al., 2015). Whether a phenotypic reversion to BZ-susceptibility by selection with MPTL is connected to the increase in heterozygosity at the codon 200 polymorphism observed in this study remains to be determined. The BZ-resistance genotypes present in the experimentally derived MTci7-12 population are equivalent to the genotypes identified in the MFie18 population, which represent the development of MPTL-resistance in the field. Genotypic characterisation of individual larvae has confirmed the BZ-resistance status of the field isolate MFie18, which was previously reported as phenotypically MPTL-resistant (Hamer et al., 2018).

In conclusion, we have shown a reduction in the number of alleles present at the *Tci-mptl-1* locus in MPTL-resistant populations when compared to their susceptible counterparts. These findings suggest that *Tci-mptl-1* was under purifying selection as a result of treatment with MPTL. This strongly implicates *Tci-mptl-1* as a mechanism of resistance to MPTL in *T. circumcincta*, which is consistent with findings in *H. contortus* (Rufener et al., 2009). Further, multiple null mutations at *Tci-mptl-1* were identified with low coverage sequencing of the full length cDNA from each population, however no mutations were shared between all resistant populations. This is similar to findings in *H. contortus* (Rufener et al., 2009) and is likely to complicate the development of resistance markers for MPTL, however further work with higher coverage sequencing may identify a common mutation in resistant isolates or, for example, a tightly linked microsatellite that could be developed as a marker. The completion of the *H. contortus* genome (https://parasite.wormbase.org/Haemonchus_contortus_prjeb506/Info/Index) and the ongoing *T. circumcincta* genome project (e.g. Choi et al., 2017) will facilitate the development of new sets of genetic markers of anthelmintic resistance. Our findings suggest that MPTL selection may alter the frequency of β-tubulin alleles and could impact on control regimens that depend upon rotations between anthelmintic classes or the use of combinations of drugs. The possibility of reversion to susceptibility is intriguing as selection with MPTL might restore susceptibility to the older broad-spectrum drench families, and warrants further investigation.

## Declaration of Competing Interest

We wish to confirm that there are no known conflicts of interest associated with this publication and there has been no significant financial support for this work that could have influenced its outcome.
