# Supplementary material for: Genotypic characterisation of monepantel resistance in historical and newly derived field strains of Teladorsagia circumcincta
Source: Int J Parasitol Drugs Drug Resist. 2019 Oct 6;11:59–69. doi: 10.1016/j.ijpddr.2019.10.002 (PMC6796645; doi:10.1016/j.ijpddr.2019.10.002)
Supplement: Multimedia component 2 [file mmc2.docx]

SUPPLEMENTARY DATA:

Supplementary Figure S1. Clustal Omega multiple sequence alignment of cDNA sequences from the *Tci-mptl-1* gene of three parental isolates and three laboratory-derived strains of *T. circumcincta*. Each sequence is the consensus derived from a minimum coverage of 3 separate sequencing runs. Identical bases are marked with a full stop (.) and gaps in the sequences are annotated with a shaded dash (-). An insertion in the *Tci-mptl-1* gene from the MTci5-13 strain introduces a stop codon, prematurely truncating the gene. An in-frame deletion of 15 bp in the *Tci-mptl-1* gene of MFie18 isolate resulting in a truncated gene with 5 fewer amino acids. The Splign program (available online at [www.ncbi.nlm.nih.gov/sutils/splign/splign.cgi](http://www.ncbi.nlm.nih.gov/sutils/splign/splign.cgi)) was used to locate exon boundaries (Kapustin *et al*., 2008).

←Exon1 Exon 2

MTci2 ATGCAGAATTTAATACTGATTCTTCTCATCAGCACCATATTTAGTCGATCAGATGCGATTTCCACAGAGGTGCCAGAACACTACCTGATAACGAATTTCATACTGTCTCGATACAACAAG 120

MTci5 .................................................................T...................................................... 120

MTci7 .........C....T..A...............................................T..........................C........T..............T... 120

MTci7_Alt .........C....T..A...............................................T..........................C........T..............T... 120

MTci11 .......................................................................A................................................ 120

MTci12 .......................................................................A................................................ 120

MTci12_Alt .......................................................................A................................................ 120

MTci13 ..............T..A...................................G.................A........T....................................... 120

MTci13_Alt ..............T..A...................................G.................A........T....................................... 120

MFie18 .................................................................T..........................C........T..............T... 120

MFie18_Alt .................................................................T..........................C........T..............T... 120

Exon 3

MTci2 GGGCTTATTCCGAAAAGACTTCAAAATGAATCGATCAAGGTGTCGTTCTCGATGGAGCTCTATCAGATTATTCAAGTGAATGAGCCACAGCAGTATCTCATGCTGAACGCCTGGATTGTT 240

MTci5 ..A..C.................................................................................................................. 240

MTci7 ..T..............G..C...........A...............................................C....................................... 240

MTci7_Alt ..T..............G..C...........A...............................................C....................................... 240

MTci11 ...................................................................................A..G...............T.............C... 240

MTci12 ...................................................................................A..G...............T.............C... 240

MTci12_Alt ...................................................................................A..G...............T.............C... 240

MTci13 ...........................................................T.......................A..................T.............C..G 240

MTci13_Alt ...........................................................T.......................A..................T.............C..G 240

MFie18 ..T..C...........G..............A..........................T....................C....................................... 240

MFie18_Alt ..T..C...........G..............A..........................T....................C....................................... 240

Exon 4 Exon 5

MTci2 GAGCGCTGGGTTGACAATCTGCTTGGATGGGATCCGGAAGAGTTCTCGAATGTCACAGAAATAATGCTGCCATACGATAACTTATGGATTCCGGACACGACGCTTTATAATTCGTTGGTC 360

MTci5 ..................................................................................................................C..... 360

MTci7 ........................................................................................................................ 360

MTci7_Alt ...................................................................................................................A.--- 357

MTci11 ............................................T.....................................................................C..... 360

MTci12 ........................................................................................................................ 360

MTci12_Alt ..............................................................C...T............................T.....A.............A.--- 357

MTci13 .........................................C..............G.....C......................................................... 360

MTci13_Alt .........................................C..............G.....C......................................................... 360

MFie18 ...................................A.................................................................................... 360

MFie18_Alt ...................................A.................................................................................... 360

MTci2 ATGGATGATCAGGACACTCGTCGTCTTCTGAACGCCAAGTTGACAACTCGAGGGAAAGACAAGGGAGCCCTCGTTGAGCTCCTCTACCCGACCATCTACAAGCTCAGCTGTTTGCTAGAT 480

MTci5 .....................................................T.................................................................. 480

MTci7 ................................................................................G....................................... 480

MTci7_Alt ------------------------------------------------------------------------------------------------------------------------ 357

MTci11 .....................................................T.................................................................. 480

MTci12 ................................................................................G....................................... 480

MTci12_Alt ------------------------------------------------------------------------------------------------------------------------ 357

MTci13 ........C..A...............T......................G..A..............A.............................................T..... 480

MTci13_Alt ........C..A...............T......................G..A..............A.............................................T..... 480

MFie18 ................................................................................G.......................A............... 480

MFie18_Alt ................................................................................G.......................A............... 480

Exon 6 Exon 7

MTci2 CTGAGATTCTTCCCCTTCGATGTGCAGACATGTAAACTAACTTTCGGCAGTTGGACGTTTGACAATACGCTCATCGACTATTTCCCTCATAATATCACTCATGCAATCGGCATCACCAAC 600

MTci5 ........................................................................................................................ 600

MTci7 ..........................A............................................................................................. 600

MTci7_Alt ------------------------------------------------------------------------------------------------------------------------ 357

MTci11 ........T............................................................................................................... 600

MTci12 ........T............................................................................................................... 600

MTci12_Alt ------------------------------------------------------------------------------------------------------------------------ 357

MTci13 ..............T...........A.......................................................................C..C.................. 600

MTci13_Alt ..............T...........A.......................................................................C..C.................. 600

MFie18 ...........T..............A........................................................T.................................... 600

MFie18_Alt ...........T..............A........................................................T.................................... 600

Exon 8

MTci2 TGCATTGACAATGAAGGATGGACAGTTCTGAAAACAACAGTGGAACGACAAGTAAATCACTACGATTGTTGTCCTAATAACTACACACTCCTGGTATTCCATCTAAATATCCAACGAAAA 720

MTci5 ..........................................................................G..C..........................G.....A......... 720

MTci7 ..........................................................................G..C...........T............T.......A......... 720

MTci7_Alt ------------------------------------------------------------------------------------------------------------------------ 357

MTci11 ......................................................................................................T................. 720

MTci12 ........................................................................................................................ 720

MTci12_Alt ------------------------------------------------------------------------------------------------------------------------ 357

MTci13 ..........................G...........C.......................T..C..C.....G..C........................T.G.....A......... 720

MTci13_Alt ..........................G...........C.......................T..C..C.....G..C........................T.G.....A......... 720

MFie18 ..........................................................................G..C...........TT...........T.......A......... 720

MFie18_Alt ..........................................................................G..C...........TT...........T.......A......... 720

Exon 9

MTci2 CCGCTCTATTACGTCATCAATCTTATTACGCCTACATCGATTATTACTCTGATCTCAATTGTTGGGTTCTTCAGTTCGTCGTCGATCAATGATCTCAGAGAGGAGAAAATCACGCTGGGA 840

MTci5 .................................................................A...................................................... 840

MTci7 ...................................G.............................A...........A.......................................... 840

MTci7_Alt ------------------------------------------------------------------------------------------------------------------------ 357

MTci11 ........................................................................................................................ 840

MTci12 ........................................................................................................................ 840

MTci12_Alt ------------------------------------------------------------------------------------------------------------------------ 357

MTci13 ..A.................C...........C..T.............................A..T........A.............................G............ 840

MTci13_Alt ..A.................C...........C..T.............................A..T........A.............................G............ 840

MFie18 ....................C............................................A...........A.......................................... 840

MFie18_Alt ....................C............................................A...........A.......................................... 840

Exon 10

MTci2 ATCACAACACTTCTGTCGATGTCTATATTAATATTCATGGTGTCCGATAAAATGCCATCAACGTCCTCTTTCATCCCTCTCATTGGATGGTTCTACACCTGTATGATACTGCTGATATCG 960

MTci5 ...........................................................G............................................................ 960

MTci7 ........G..................................................G.....G..C..............A........T........................... 960

MTci7_Alt ------------------------------------------------------------------------------------------------------------------------ 357

MTci11 ..................................................................................................A...........A......... 960

MTci12 ..................................................................................................A...........A......... 960

MTci12_Alt ------------------------------------------------------------------------------------------------------------------------ 357

MTci13 .............................G.............................G.....G..C..............A.................................... 960

MTci13_Alt .............................G.............................G.....G..C..............A.TGAAAGCTGTTGGATTTTTTTTTGCCTAA------ 960

MFie18 ...........................................................G.....G..C..............A........T........................... 960

MFie18_Alt ...........................................................G.....G..C..............A........T........................... 960

Exon 11

MTci2 TTCTCAACGTTGGCCGCTTCCATGGTCATATACGTTCAGAAGCAGGGTATTCTTGGCAAACCACCATGTCGAAAAACGATGCGCTGGGCGCGTCTCGTAGCACGATGTGTCCGAATGGAG 1080

MTci5 ...................................G........................................................A........G.................. 1080

MTci7 ...................................G........................................................A.....G..G.................. 1080

MTci7_Alt ------------------------------------------------------------------------------------------------------------------------ 357

MTci11 ............................................................................................A........G........T......... 1080

MTci12 ............................................................................................A.....G..G.................. 1080

MTci12_Alt ------------------------------------------------------------------------------------------------------------------------ 357

MTci13 ..........................T........G.......................G.............................A..A..G.....G.................. 1080

MTci13_Alt ------------------------------------------------------------------------------------------------------------------------ 964

MFie18 ...................................A........................................................A........G.................. 1080

MFie18_Alt ...................................A........................................................A........G.................. 1080

Exon 12

MTci2 ATGCCGCTTCTCATGAAACAGGCCTATGCGCAAAAGGCTCGAGAGGACAAATTGAGGCGCGCTCAAGATGGACGAAAACAGAGTCTATGGCAGCGCGTGTATAGGTTAGCTAGAGAACAG 1200

MTci5 .................G...................................................................................C.................. 1200

MTci7 .................G...................................................................................C.................. 1200

MTci7_Alt ------------------------------------------------------------------------------------------------------------------------ 357

MTci11 ...........................................................T...........T........A.................C..................... 1200

MTci12 ....................A.................C..................................................................A.......G...... 1200

MTci12_Alt ------------------------------------------------------------------------------------------------------------------------ 357

MTci13 .................G......................................A............................................C...A.............. 1200

MTci13_Alt ------------------------------------------------------------------------------------------------------------------------ 964

MFie18 .................G...................................................................................C...A.............. 1200

MFie18_Alt .....................................................................................................C...A.............. 1200

Exon 13

MTci2 GGACAGATTAGAAAGCAATCGAACACGTTGCCTAAAGTGAATGGAGTCGGTGGAATTGGGAGCACATCGTCACCGGATATTCAACAATTACAAGTGCCAAAGAAGAGCTGCACAATCAAC 1320

MTci5 .................................................................G...................................................... 1320

MTci7 .................................................................G...........C........GC................................ 1320

MTci7_Alt ------------------------------------------------------------------------------------------------------------------------ 357

MTci11 ..............................................................................................................T......... 1320

MTci12 ........................................................................................................................ 1320

MTci12_Alt ------------------------------------------------------------------------------------------------------------------------ 357

MTci13 ........C..G.....................................................G..C........C........GC................................ 1320

MTci13_Alt ------------------------------------------------------------------------------------------------------------------------ 964

MFie18 .................................................................G...........C.....G..GC................................ 1320

MFie18_Alt .................................................................G...........C.....G..GC................................ 1320

Exon 14

MTci2 ACGGACGTCACGTGTATCAATGAGCAGCGTGACACAAGCGCATTGGTGGAATTTTCGAACATGTCGGATGAGGACAACTCGTCCTTCCCGGACATTGACTTTGCAGCTGCGACTCCAGCC 1440

MTci5 ........................................................................................................................ 1440

MTci7 ..............................................................................................................A......... 1440

MTci7_Alt ------------------------------------------------------------------------------------------------------------------------ 357

MTci11 .................................................................................................................C...... 1440

MTci12 ..............................................................................................................A......... 1440

MTci12_Alt ------------------------------------------------------------------------------------------------------------------------ 357

MTci13 ..............................................................................................................A......... 1440

MTci13_Alt ------------------------------------------------------------------------------------------------------------------------ 964

MFie18 ..............................................................................................................A......... 1440

MFie18_Alt ..............---------------.................................................................................A......... 1365

Exon 15

MTci2 ACTCCAGTGTCCAAATTCCACAGTCTGCACAAGATGAGCACGTGCGCATCGCTAGACAGTATGATTCGCAATGTGGACATGTCAGTGACGTCACCGCGAACGATTCAACGGAATCTTGCC 1560

MTci5 ..C.................T...........A....................G.....C.................T.......................A.....G............ 1560

MTci7 ................................A..........................................................................G............ 1560

MTci7_Alt ------------------------------------------------------------------------------------------------------------------------ 357

MTci11 ...........T............................................................................................A............... 1560

MTci12 ................................A....................................................................................... 1560

MTci12_Alt ------------------------------------------------------------------------------------------------------------------------ 357

MTci13 ................................A..........................................................................G............ 1560

MTci13_Alt ------------------------------------------------------------------------------------------------------------------------ 964

MFie18 ................................A............................................T.......................A.....G............ 1560

MFie18_Alt ................................A............................................T.......................A.....G............ 1365

MTci2 GAACTGGAATACGATTGGTTGGCAGCCGTAATTGAACGGATTTTTTTGATTTTCTTTATTATTGTTTTCTTGTTGACTTCTGTTGGTATTAATTGTATTGGTTTGTATTATTGGTATGTC 1680

MTci5 ..G.....................................................C............................................................... 1680

MTci7 ..G..................................................................................................................... 1680

MTci7_Alt ------------------------------------------------------------------------------------------------------------------------ 357

MTci11 .............................................C.........................................................................T 1680

MTci12 .............................................C..........C............................................................... 1680

MTci12_Alt ------------------------------------------------------------------------------------------------------------------------ 357

MTci13 ..G..........................................C..........C............................................................... 1680

MTci13_Alt ------------------------------------------------------------------------------------------------------------------------ 964

MFie18 ..G..................................................................................................................... 1680

MFie18_Alt ..G..................................................................................................................... 1365

MTci2 GCACAAAATGACCCTCACTTTGGTTTCCCATCATAA 1716

MTci5 ........................C........... 1716

MTci7 ........................C........... 1716

MTci7_Alt ------------------------------------ 357

MTci11 ........................C........... 1716

MTci12 .................................... 1716

MTci12_Alt ------------------------------------ 357

MTci13 ........................C........... 1716

MTci13_Alt ------------------------------------ 954

MFie18 .................................... 1716

MFie18_Alt .................................... 1701

Supplementary Figure S2. Comparisons of predicted *Tci-mptl-1* amino acid sequences generated from seven populations of *T. circumcincta* and aligned with the multiple sequence alignment program Clustal-omega. Identical residues are represented with . gaps in the sequence are annotated with – and stop codons are represented by *****. Regions conserved between nAChR α subunits are highlighted in blue (export signal), black (loops) or red (trans-membrane domains; TMD 1-4).

**Export Signal**

MTci2 MQNLILILLISTIFSRSDAISTEVPEHYLITNFILSRYNKGLIPKRLQNESIKVSFSMEL 60

MTci5 ............................................................ 60

MTci7 ............................................................ 60

MTci7_Alt ............................................................ 60

MTci2-11 ............................................................ 60

MTci7-12 ............................................................ 60

MTci7-12_Alt ............................................................ 60

MTci5-13 .................E.......................................... 60

MTci5-13_Alt .................E.......................................... 60

MFie18 ............................................................ 60

MFie18_Alt ............................................................ 60

**D-loop A-loop**

MTci2 YQIIQVNEPQQYLMLNAWIVERWVDNLLGWDPEEFSNVTEIMLPYDNLWIPDTTLYNSLV 120

MTci5 ............................................................ 120

MTci7 ............................................................ 120

MTci7_Alt ..........................................................*****- 118

MTci2-11 ............................................................ 120

MTci7-12 ............................................................ 120

MTci7-12_Alt ..........................................................*****- 118

MTci5-13 .................................D.......................... 120

MTci5-13_Alt .................................D.......................... 120

MFie18 ............................................................ 120

MFie18_Alt ............................................................ 120

**E-loop Cys-Loop B-loop**

MTci2 MDDQDTRRLLNAKLTTRGKDKGALVELLYPTIYKLSCLLDLRFFPFDVQTCKLTFGSWTF 180

MTci5 ............................................................ 180

MTci7 ............................................................ 180

MTci7_Alt ------------------------------------------------------------ 118

MTci2-11 ............................................................ 180

MTci7-12 ............................................................ 180

MTci7-12_Alt ------------------------------------------------------------ 118

MTci5-13 ............................................................ 180

MTci5-13_Alt ............................................................ 180

MFie18 ............................................................ 180

MFie18_Alt ............................................................ 180

**F-loop C-loop**

MTci2 DNTLIDYFPHNITHAIGITNCIDNEGWTVLKTTVERQVNHYDCCPNNYTLLVFHLNIQRK 240

MTci5 ............................................................ 240

MTci7 ............................................................ 240

MTci7_Alt ------------------------------------------------------------ 118

MTci2-11 ............................................................ 240

MTci7-12 ............................................................ 240

MTci7-12_Alt ------------------------------------------------------------ 118

MTci5-13 ............................................................ 240

MTci5-13_Alt ............................................................ 240

MFie18 ............................................................ 240

MFie18_Alt ............................................................ 240

**TMD 1 TMD 2**

MTci2 PLYYVINLITPTSIITLISIVGFFSSSSINDLREEKITLGITTLLSMSILIFMVSDKMPS 300

MTci5 ............................................................ 300

MTci7 ............................................................ 300

MTci7_Alt ------------------------------------------------------------ 118

MTci2-11 ............................................................ 300

MTci7-12 ............................................................ 300

MTci7-12_Alt ------------------------------------------------------------ 118

MTci5-13 ............................................................ 300

MTci5-13_Alt ............................................................ 300

MFie18 ............................................................ 300

MFie18_Alt ............................................................ 300

**TMD 3**

MTci2 TSSFIPLIGWFYTCMILLISFSTLAASMVIYVQKQGILGKPPCRKTMRWARLVARCVRME 360

MTci5 ............................................................ 360

MTci7 ............................................................ 360

MTci7_Alt ------------------------------------------------------------ 118

MTci2-11 ............................................................ 360

MTci7-12 ............................................................ 360

MTci7-12_Alt ------------------------------------------------------------ 118

MTci5-13 ............................................................ 360

MTci5-13_Alt ........VKAVGFFFA*------------------------------------------ 317

MFie18 ............................................................ 360

MFie18_Alt ............................................................ 360

MTci2 MPLLMKQAYAQKAREDKLRRAQDGRKQSLWQRVYRLAREQGQIRKQSNTLPKVNGVGGIG 420

MTci5 ............................................................ 420

MTci7 ............................................................ 420

MTci7_Alt ------------------------------------------------------------ 118

MTci2-11 ............................................................ 420

MTci7-12 ............................................................ 420

MTci7-12_Alt ------------------------------------------------------------ 118

MTci5-13 ............................................................ 420

MTci5-13_Alt ------------------------------------------------------------ 317

MFie18 ............................................................ 420

MFie18_Alt ............................................................ 420

MTci2 STSSPDIQQLQVPKKSCTINTDVTCINEQRDTSALVEFSNMSDEDNSSFPDIDFAAATPA 480

MTci5 ............................................................ 480

MTci7 ............................................................ 480

MTci7_Alt ------------------------------------------------------------ 118

MTci2-11 ............................................................ 480

MTci7-12 ............................................................ 480

MTci7-12_Alt ------------------------------------------------------------ 118

MTci5-13 ............................................................ 480

MTci5-13_Alt ------------------------------------------------------------ 317

MFie18 ............................................................ 480

MFie18_Alt .............................-----.......................... 475

**TMD 4**

MTci2 TPVSKFHSLHKMSTCASLDSMIRNVDMSVTSPRTIQRNLAELEYDWLAAVIERIFLIFFI 540

MTci5 ............................................................ 540

MTci7 ............................................................ 540

MTci7_Alt ------------------------------------------------------------ 118

MTci2-11 ............................................................ 540

MTci7-12 ............................................................ 540

MTci7-12_Alt ------------------------------------------------------------ 118

MTci5-13 ............................................................ 540

MTci5-13_Alt ------------------------------------------------------------ 317

MFie18 ............................................................ 540

MFie18_Alt ............................................................ 535

MTci2 IVFLLTSVGINCIGLYYWYVAQNDPHFGFPS* 571

MTci5 ............................L..* 571

MTci7 ............................L..* 571

MTci7_Alt ------------------------------- 118

MTci2-11 ............................L..* 571

MTci7-12 ...............................* 571

MTci7-12_Alt ------------------------------- 118

MTci5-13 ............................L..* 571

MTci5-13_Alt ------------------------------- 317

MFie18 ...............................* 571

MFie18_Alt ...............................* 566

Supplementary Table S3. Percentage identity between the *Tci-mptl-1* gene sequences in seven UK populations of *T. circumcincta*. Clustal Omega alignment was conducted and the percentage identity between the cDNA sequences (below the diagonal) and the translated amino acid sequences (above the diagonal) were calculated between the MPTL-susceptible isolates (MTci2, MTci5 and MTci7), the derived MPTL-resistant strains (MTci2-11, MTci5-13 and MTci7-12) and a MPTL-resistant UK field isolate (MFie18).

| Pop^n^ | MTci  2 | MTci  5 | MTci  7 | MTci  7_Alt | MTci  2-11 | MTci  7-12 | MTci  7-12_Alt | MTci  5-13 | MTci  5-13_Alt | MFie  18 | MFie  18_Alt | Amino Acid Identity (%) |
| --- | --- | --- | --- | --- | --- | --- | --- | --- | --- | --- | --- | --- |
| MTci2 |  | 99.82 | 99.82 | 100 | 99.82 | 100 | 100 | 99.47 | 98.40 | 100 | 100 |  |
| MTci5 | 98.31 |  | 100 | 100 | 100 | 99.82 | 100 | 99.65 | 98.40 | 99.82 | 99.82 |  |
| MTci7 | 97.44 | 97.73 |  | 100 | 100 | 99.82 | 100 | 99.65 | 98.40 | 99.82 | 99.82 |  |
| MTci7_Alt | 96.36 | 96.08 | 99.72 |  | 100 | 100 | 100 | 98.31 | 98.31 | 100 | 100 |  |
| MTci2-11 | 98.48 | 97.38 | 96.39 | 94.40 |  | 99.82 | 100 | 99.65 | 98.40 | 99.82 | 99.82 |  |
| MTci7-12 | 98.60 | 97.55 | 96.85 | 94.96 | 98.43 |  | 100 | 99.47 | 98.40 | 100 | 100 |  |
| MTci7-12_Alt | 97.20 | 96.08 | 93.84 | 94.12 | 98.04 | 98.60 |  | 98.31 | 98.31 | 100 | 100 |  |
| MTci5-13 | 95.80 | 96.21 | 96.45 | 93,84 | 95.40 | 95.80 | 96.36 |  | 99.04 | 99.47 | 99.47 |  |
| MTci5-13_Alt | 92.45 | 92.56 | 92.56 | 93.84 | 92.66 | 92.56 | 96.36 | 97.38 |  | 98.40 | 98.40 |  |
| MFie18 | 97.32 | 97.49 | 99.72 | 97.76 | 97.15 | 96.68 | 94.12 | 96.04 | 92.24 |  | 100 |  |
| MFie18_Alt | 97.30 | 97.47 | 99.71 | 97.76 | 96.12 | 96.65 | 94.12 | 96.00 | 92.24 | 100 |  |  |
|  | cDNA Identity (%) | | | | | | | | | | |  |

Supplementary Table S4. Population genetic data for ten microsatellite markers in individual L_3_ from nine populations of *T. circumcincta* (i.e. 2760 genotypes contribute to the data in the table). *N*_0_, number of worms which failed to give an amplification product for a particular marker (apparent null homozygotes). Arlequin ver 3.5.2.2 software was used to determine the *H*_e_ (expected heterozygosity, Nei’s unbiased), *H*_o_ (observed heterozygosity) and *F*_IS_ inbreeding coefficient. *P*-values < 0.005 indicate a significant deviation from Hardy-Weinberg equilibrium after Bonferroni correction. *A*, number of alleles per locus per population; *N*_f_, estimated null allele frequency. Mean values for all loci are also included.

| Population (n) | MTG15 | MTG67 | MTG68 | MTG73 | Tc2066 | Tc2467 | Tc4504 | Tc7989 | Tc13604 | Tc22274 | All Loci |
| --- | --- | --- | --- | --- | --- | --- | --- | --- | --- | --- | --- |
| MTci2 (31) |  |  |  |  |  |  |  |  |  |  |  |
| *N*_0_ | 5 | 4 | 4 | 0 | 14 | 1 | 11 | 14 | 20 | 7 |  |
| *H*_e_ | 0.853 | 0.744 | 0.820 | 0.317 | 0.752 | 0.906 | 0.885 | 0.950 | 0.805 | 0.902 | 0.793 |
| *H_o_* | 0.769 | 0.481 | 0.778 | 0.387 | 0.294 | 0.900 | 0.600 | 0.706 | 0.364 | 0.750 | 0.603 |
| *P*-value | 0.003 | 0.002 | 0.325 | 0.558 | *<0.001* | 0.002 | *<0.001* | 0.004 | 0.003 | *<0.001* |  |
| *F*_IS_ | 0.100 | 0.357 | 0.052 | -0.224 | 0.616 | 0.006 | 0.327 | 0.263 | 0.560 | 0.172 | 0.223 |
| *A* | 9 | 9 | 7 | 2 | 6 | 13 | 9 | 17 | 7 | 15 | 9.4 |
| *N*_f_ | 0.238 | 0.296 | 0.200 | 0.000 | 0.631 | 0.068 | 0.475 | 0.529 | 0.754 | 0.313 |  |
| MTci5 (31) |  |  |  |  |  |  |  |  |  |  |  |
| *N*_0_ | 12 | 5 | 6 | 2 | 8 | 15 | 3 | 2 | 8 | 10 |  |
| *H*_e_ | 0.929 | 0.796 | 0.637 | 0.257 | 0.799 | 0.897 | 0.886 | 0.944 | 0.919 | 0.930 | 0.799 |
| *H_o_* | 0.737 | 0.538 | 0.720 | 0.207 | 0.174 | 0.500 | 0.714 | 0.793 | 0.391 | 0.714 | 0.549 |
| *P*-value | *<0.001* | *<0.001* | 0.735 | 0.113 | *<0.001* | *<0.001* | 0.002 | 0.004 | *<0.001* | *<0.001* |  |
| *F*_IS_ | 0.211 | 0.328 | -0.134 | 0.198 | 0.786 | 0.451 | 0.196 | 0.162 | 0.580 | 0.237 | 0.302 |
| *A* | 13 | 8 | 7 | 6 | 7 | 11 | 13 | 23 | 14 | 15 | 11.7 |
| *N*_f_ | 0.465 | 0.318 | 0.262 | 0.222 | 0.538 | 0.609 | 0.196 | 0.147 | 0.473 | 0.395 |  |
| MTci7 (31) |  |  |  |  |  |  |  |  |  |  |  |
| *N*_0_ | 2 | 4 | 2 | 3 | 9 | 10 | 7 | 1 | 7 | 9 |  |
| *H*_e_ | 0.852 | 0.827 | 0.812 | 0.254 | 0.833 | 0.907 | 0.878 | 0.907 | 0.887 | 0.930 | 0.809 |
| *H_o_* | 0.759 | 0.630 | 0.724 | 0.214 | 0.455 | 0.476 | 0.625 | 0.833 | 0.375 | 0.773 | 0.586 |
| *P*-value | *<0.001* | 0.006 | 0.295 | 0.442 | *<0.001* | *<0.001* | *<0.001* | *<0.001* | *<0.001* | *<0.001* |  |
| *F*_IS_ | 0.112 | 0.242 | 0.110 | 0.158 | 0.460 | 0.481 | 0.292 | 0.083 | 0.582 | 0.173 | 0.269 |
| *A* | 13 | 13 | 7 | 3 | 9 | 12 | 9 | 18 | 13 | 16 | 11.3 |
| *N*_f_ | 0.137 | 0.253 | 0.153 | 0.268 | 0.464 | 0.490 | 0.354 | 0.085 | 0.451 | 0.367 |  |
| MTci2-11 (31) |  |  |  |  |  |  |  |  |  |  |  |
| *N*_0_ | 9 | 4 | 3 | 2 | 11 | 5 | 6 | 7 | 4 | 7 |  |
| *H*_e_ | 0.680 | 0.752 | 0.636 | 0.068 | 0.786 | 0.867 | 0.809 | 0.837 | 0.864 | 0.912 | 0.721 |
| *H_o_* | 0.500 | 0.556 | 0.393 | 0.069 | 0.200 | 0.808 | 0.520 | 0.375 | 0.519 | 0.625 | 0.456 |
| *P*-value | 0.003 | 0.054 | 0.004 | 1.000 | *<0.001* | 0.011 | 0.003 | *<0.001* | *<0.001* | *<0.001* |  |
| *F*_IS_ | 0.269 | 0.265 | 0.386 | -0.018 | 0.750 | 0.070 | 0.362 | 0.557 | 0.405 | 0.320 | 0.337 |
| *A* | 10 | 6 | 4 | 2 | 6 | 12 | 6 | 9 | 10 | 14 | 7.9 |
| *N*_f_ | 0.434 | 0.273 | 0.276 | 0.234 | 0.594 | 0.230 | 0.355 | 0.444 | 0.308 | 0.393 |  |
| MTci7-12 (31) |  |  |  |  |  |  |  |  |  |  |  |
| *N*_0_ | 7 | 3 | 4 | 3 | 16 | 7 | 18 | 3 | 4 | 2 |  |
| *H*_e_ | 0.915 | 0.690 | 0.656 | 0.398 | 0.851 | 0.874 | 0.871 | 0.883 | 0.894 | 0.896 | 0.793 |
| *H_o_* | 0.667 | 0.464 | 0.667 | 0.286 | 0.200 | 0.667 | 0.846 | 0.750 | 0.815 | 0.759 | 0.612 |
| *P*-value | 0.003 | 0.010 | 0.615 | 0.063 | *<0.001* | 0.002 | 0.053 | 0.180 | 0.556 | 0.007 |  |
| *F*_IS_ | 0.276 | 0.331 | -0.016 | 0.286 | 0.771 | 0.241 | 0.029 | 0.153 | 0.090 | 0.156 | 0.232 |
| *A* | 13 | 7 | 5 | 4 | 7 | 12 | 9 | 16 | 13 | 17 | 10.3 |
| *N*_f_ | 0.343 | 0.265 | 0.209 | 0.270 | 0.703 | 0.338 | 0.611 | 0.183 | 0.192 | 0.142 |  |
| MTci5-13 (31) |  |  |  |  |  |  |  |  |  |  |  |
| *N*_0_ | 4 | 11 | 4 | 5 | 11 | 9 | 20 | 4 | 3 | 1 |  |
| *H*_e_ | 0.786 | 0.851 | 0.681 | 0.148 | 0.606 | 0.793 | 0.818 | 0.922 | 0.823 | 0.797 | 0.723 |
| *H_o_* | 0.593 | 0.600 | 0.519 | 0.077 | 0.150 | 0.636 | 0.818 | 0.889 | 0.571 | 0.733 | 0.559 |
| *P*-value | 0.029 | 0.005 | 0.016 | 0.040 | *<0.001* | *<0.001* | *<0.001* | 0.737 | *<0.001* | 0.194 |  |
| *F*_IS_ | 0.250 | 0.301 | 0.242 | 0.485 | 0.757 | 0.201 | 0.000 | 0.036 | 0.310 | 0.081 | 0.266 |
| *A* | 7 | 7 | 5 | 3 | 5 | 10 | 6 | 18 | 11 | 16 | 8.8 |
| *N*_f_ | 0.261 | 0.474 | 0.277 | 0.395 | 0.595 | 0.404 | 0.676 | 0.166 | 0.247 | 0.083 |  |
| MFie18 Pre (29) |  |  |  |  |  |  |  |  |  |  |  |
| *N*_0_ | 16 | 3 | 8 | 4 | 13 | 18 | 22 | 4 | 9 | 12 |  |
| *H*_e_ | 0.857 | 0.748 | 0.788 | 0.515 | 0.840 | 0.899 | 0.800 | 0.949 | 0.922 | 0.906 | 0.822 |
| *H_o_* | 0.429 | 0.370 | 0.273 | 0.308 | 0.235 | 0.000 | 0.000 | 0.538 | 0.381 | 0.611 | 0.315 |
| *P*-value | *<0.001* | *<0.001* | *<0.001* | 0.006 | *<0.001* | *<0.001* | *<0.001* | *<0.001* | *<0.001* | 0.002 |  |
| *F*_IS_ | 0.509 | 0.509 | 0.659 | 0.407 | 0.726 | 1.000 | 1.000 | 0.438 | 0.593 | 0.332 | 0.617 |
| *A* | 12 | 8 | 5 | 4 | 8 | 9 | 5 | 24 | 13 | 12 | 10.0 |
| *N*_f_ | 0.660 | 0.318 | 0.507 | 0.328 | 0.639 | 0.797 | 0.864 | 0.318 | 0.507 | 0.510 |  |
| MFie18 Post (30) |  |  |  |  |  |  |  |  |  |  |  |
| *N*_0_ | 8 | 7 | 7 | 8 | 15 | 18 | 20 | 8 | 13 | 17 |  |
| *H*_e_ | 0.790 | 0.775 | 0.766 | 0.559 | 0.710 | 0.859 | 0.795 | 0.912 | 0.888 | 0.914 | 0.797 |
| *H_o_* | 0.409 | 0.391 | 0.348 | 0.364 | 0.200 | 0.083 | 0.100 | 0.455 | 0.176 | 0.154 | 0.268 |
| *P*-value | *<0.001* | *<0.001* | *<0.001* | 0.064 | *<0.001* | *<0.001* | *<0.001* | *<0.001* | *<0.001* | *<0.001* |  |
| *F*_IS_ | 0.488 | 0.501 | 0.552 | 0.355 | 0.725 | 0.907 | 0.880 | 0.508 | 0.806 | 0.837 | 0.656 |
| *A* | 8 | 7 | 5 | 3 | 5 | 8 | 6 | 20 | 13 | 9 | 8.4 |
| *N*_f_ | 0.459 | 0.434 | 0.450 | 0.448 | 0.686 | 0.779 | 0.813 | 0.454 | 0.659 | 0.746 |  |
| UK049L09 Pre (31) |  |  |  |  |  |  |  |  |  |  |  |
| *N*_0_ | 2 | 0 | 2 | 0 | 12 | 8 | 12 | 4 | 6 | 9 |  |
| *H*_e_ | 0.915 | 0.809 | 0.776 | 0.506 | 0.814 | 0.922 | 0.900 | 0.943 | 0.923 | 0.916 | 0.842 |
| *H_o_* | 0.828 | 0.677 | 0.621 | 0.387 | 0.263 | 0.478 | 0.053 | 0.889 | 0.440 | 0.455 | 0.509 |
| *P*-value | 0.013 | 0.032 | 0.051 | 0.057 | *<0.001* | *<0.001* | *<0.001* | 0.087 | *<0.001* | *<0.001* |  |
| *F*_IS_ | 0.097 | 0.165 | 0.203 | 0.238 | 0.683 | 0.487 | 0.943 | 0.058 | 0.529 | 0.510 | 0.391 |
| *A* | 16 | 10 | 7 | 4 | 8 | 14 | 10 | 23 | 18 | 12 | 12.2 |
| *N*_f_ | 0.124 | 0.060 | 0.174 | 0.105 | 0.598 | 0.441 | 0.669 | 0.169 | 0.404 | 0.474 |  |

Supplementary Figure S5. An example of gDNA sequence data from amplicons that include exons 7 and 8 of *Tci-mptl-1* aligned with SeqMan Pro™ software (DNASTAR^®^ Lasergene^®^ v15). Polymorphic bases (arrowed) were recorded and a genotype number was allocated to each combination of polymorphisms. The translated amino acid sequence is show in green. Each larva was DNA sequenced in both the sense and antisense directions to ensure data account for sequencing errors or gaps.


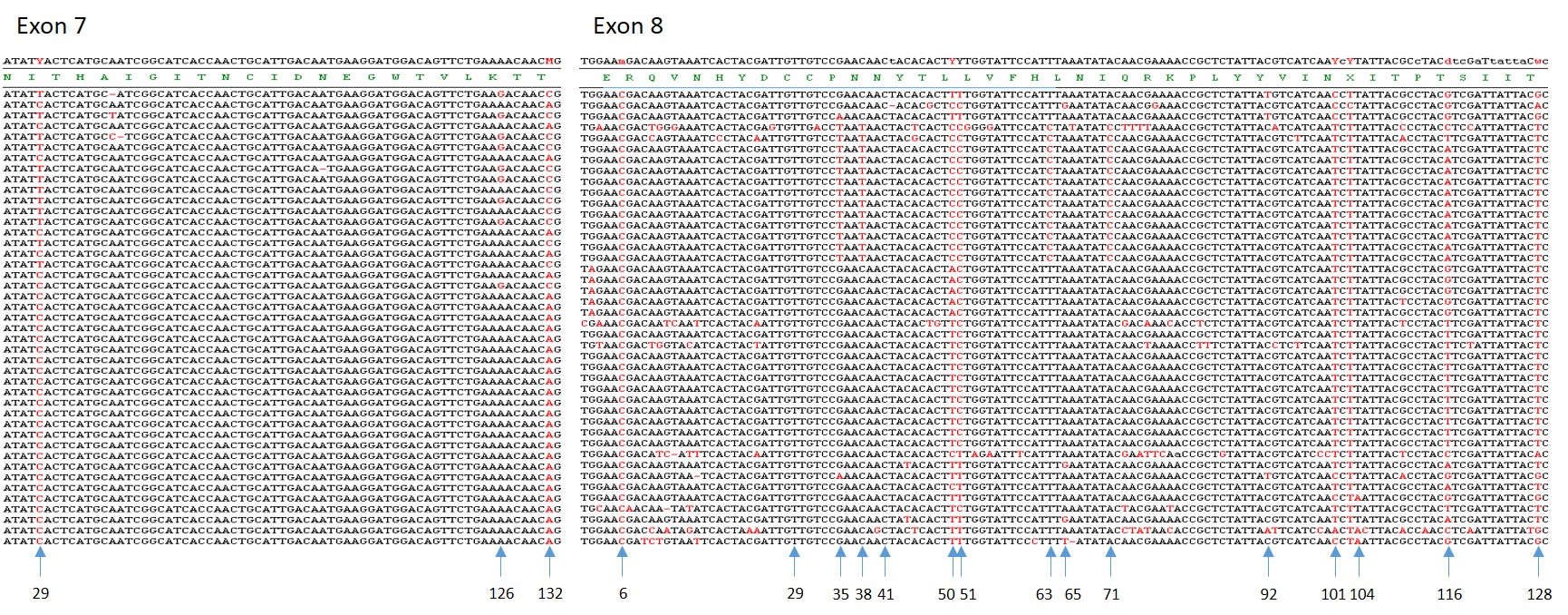


Supplementary Table S6. Prevalence of mutations at positions 167, 198 and 200 of β-tubulin isotype 1 gene in individual larvae from nine different populations of *T. circumcincta*. The proportion of each genotype is reported and the actual numbers of larvae is displayed in parenthesis [squared brackets].

| **Population**  (Phenotype) | **Position 167** | | |  | **Position 198** | | |  | **Position 200** | | |
| --- | --- | --- | --- | --- | --- | --- | --- | --- | --- | --- | --- |
|  | **Phe/Phe**  **(S/S)** | **Phe/Tyr**  **(S/r)** | **Tyr/Tyr**  **(r/r)** |  | **Glu/Glu**  **(S/S)** | **Glu/Ala**  **(S/r)** | **Ala/Ala**  **(r/r)** |  | **Phe/Phe**  **(S/S)** | **Phe/Tyr**  **(S/r)** | **Tyr/Tyr**  **(r/r)** |
| MTci2  (BZ-s, mptl-s) | 100 %  [28/28] | 0 % | 0 % |  | 100 %  [30/30] | 0 % | 0 % |  | 66.7 % [20/30] | 23.3 % [7/30] | 10.0 % [3/30] |
| MTci2-11  (mptl -r) | 100 % [27/27] | 0 % | 0 % |  | 100 % [27/27] | 0 % | 0 % |  | 77.8 % [21/27] | 22.2 % [6/27] | 0 % |
| MTci5  (BZ-r, mptl -s) | 96.6 % [28/29] | 3.4 % [1/29] | 0 % |  | 100 % [30/30] | 0 % | 0 % |  | 23.3 % [7/30] | 53.3 % [16/30] | 23.3 % [7/30] |
| MTci5-13  (mptl -r) | 100 %  [29/29] | 0 % | 0 % |  | 100 %  [26/26] | 0 % | 0 % |  | 15.4 % [4/26] | 69.2 % [18/26] | 15.4 % [4/26] |
| MTci7  (BZ-r, mptl-s) | 100 %  [31/31] | 0 % | 0 % |  | 100 % [30/30] | 0 % | 0 % |  | 3.3 % [1/30] | 30.0 % [9/30] | 66.7 %  [20/30] |
| MTci7-12  (mptl -r) | 100 % [31/31] | 0 % | 0 % |  | 100 % [28/28] | 0 % | 0 % |  | 10.7 % [3/28] | 42.9 % [12/28] | 46.4 % [13/28] |
| MFie18 Pre  (mptl-r) | 100 % [29/29] | 0 % | 0 % |  | 100 % [29/29] | 0 % | 0 % |  | 10.3 % [3/29] | 44.8 % [13/29] | 44.8 % [13/29] |
| MFie18 Post  (mptl-r) | 100 % [30/30] | 0 % | 0 % |  | 100 % [29/29] | 0 % | 0 % |  | 6.9 % [2/29] | 41.4 % [12/29] | 51.7 % [15/29] |
| UK049L09  (BZ-r, mptl-s) | 100 % [31/31] | 0 % | 0 % |  | 100 % [26/26] | 0 % | 0 % |  | 7.7 % [2/26] | 30.8 % [8/26] | 61.5 % [16/26] |
